# Molecular consequences of SARS-CoV-2 liver tropism

**DOI:** 10.1038/s42255-022-00552-6

**Published:** 2022-03-28

**Authors:** Nicola Wanner, Geoffroy Andrieux, Pau Badia-i-Mompel, Carolin Edler, Susanne Pfefferle, Maja T. Lindenmeyer, Christian Schmidt-Lauber, Jan Czogalla, Milagros N. Wong, Yusuke Okabayashi, Fabian Braun, Marc Lütgehetmann, Elisabeth Meister, Shun Lu, Maria L. M. Noriega, Thomas Günther, Adam Grundhoff, Nicole Fischer, Hanna Bräuninger, Diana Lindner, Dirk Westermann, Fabian Haas, Kevin Roedl, Stefan Kluge, Marylyn M. Addo, Samuel Huber, Ansgar W. Lohse, Jochen Reiser, Benjamin Ondruschka, Jan P. Sperhake, Julio Saez-Rodriguez, Melanie Boerries, Salim S. Hayek, Martin Aepfelbacher, Pietro Scaturro, Victor G. Puelles, Tobias B. Huber

**Affiliations:** 1 III. Department of Medicine, University Medical Center Hamburg-Eppendorf, Hamburg, Germany; 2 Institute of Medical Bioinformatics and Systems Medicine, Medical Center-University of Freiburg, Faculty of Medicine, University of Freiburg, Freiburg, Germany; 3 Institute for Computational Biomedicine, Faculty of Medicine, Heidelberg University and Heidelberg University Hospital, BioQuant, Heidelberg, Germany; 4 Institute of Legal Medicine, University Medical Center Hamburg-Eppendorf, Hamburg, Germany; 5 Institute of Medical Microbiology, Virology and Hygiene, University Medical Center Hamburg-Eppendorf, Hamburg, Germany; 6 Institute of Pathology, University Medical Center Hamburg-Eppendorf, Hamburg, Germany; 7 Leibniz Institute for Experimental Virology, Hamburg, Germany; 8 Department of Cardiology, University Heart and Vascular Centre Hamburg, University Medical Center Hamburg-Eppendorf, Hamburg, Germany; 9 DZHK (German Centre for Cardiovascular Research), Partner Site Hamburg/Kiel/Lübeck, Hamburg, Germany; 10 Department of Intensive Care Medicine, University Medical Center Hamburg-Eppendorf, Hamburg, Germany; 11 I. Department of Medicine, University Medical Hospital Hamburg-Eppendorf, Hamburg, Germany; 12 German Center for Infection Research, Hamburg-Lübeck-Borstel-Riems, Hamburg, Germany; 13 Department of Medicine, Rush University Medical Center, Chicago, IL USA; 14 German Cancer Consortium and German Cancer Research Center, Partner Site Freiburg, Freiburg, Germany; 15 Division of Cardiology, Department of Internal Medicine, University of Michigan, Ann Arbor, MI USA

**Keywords:** SARS-CoV-2, Liver, Metabolism

## Abstract

Extrapulmonary manifestations of COVID-19 have gained attention due to their links to clinical outcomes and their potential long-term sequelae^1^. Severe acute respiratory syndrome coronavirus 2 (SARS-CoV-2) displays tropism towards several organs, including the heart and kidney. Whether it also directly affects the liver has been debated^2,3^. Here we provide clinical, histopathological, molecular and bioinformatic evidence for the hepatic tropism of SARS-CoV-2. We find that liver injury, indicated by a high frequency of abnormal liver function tests, is a common clinical feature of COVID-19 in two independent cohorts of patients with COVID-19 requiring hospitalization. Using autopsy samples obtained from a third patient cohort, we provide multiple levels of evidence for SARS-CoV-2 liver tropism, including viral RNA detection in 69% of autopsy liver specimens, and successful isolation of infectious SARS-CoV-2 from liver tissue postmortem. Furthermore, we identify transcription-, proteomic- and transcription factor-based activity profiles in hepatic autopsy samples, revealing similarities to the signatures associated with multiple other viral infections of the human liver. Together, we provide a comprehensive multimodal analysis of SARS-CoV-2 liver tropism, which increases our understanding of the molecular consequences of severe COVID-19 and could be useful for the identification of organ-specific pharmacological targets.

## Abstract (web summary)

Wanner et al. demonstrate SARS-CoV-2 liver tropism and identify transcriptional and proteomic profiles of SARS-CoV-2 liver samples that show similarities to previously characterized hepatotropic viruses.

## Main

**Fig. 1:**
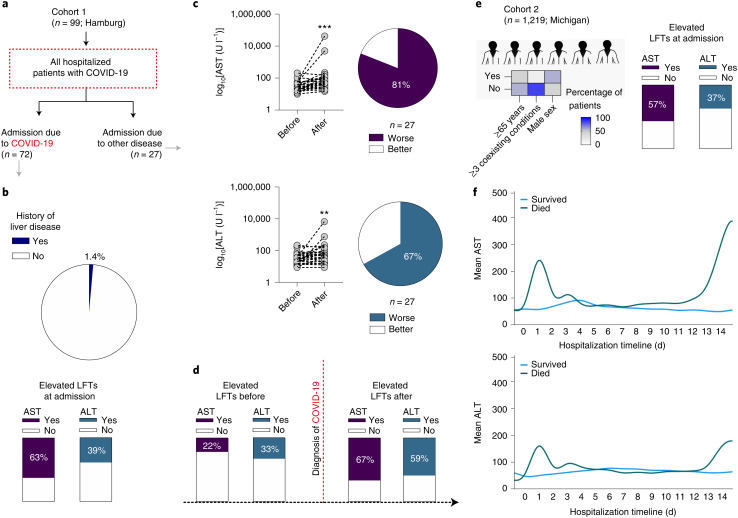
Elevated LFTs among patients with COVID-19. **a**, Overview of cohort 1. *n* = 99 patients required hospitalization due to COVID-19 (Germany). *n* = 72 were admitted due to moderate/severe COVID-19 and *n* = 27 acquired COVID-19 during their hospital stay. **b**, Only 1.4% of patients admitted due to COVID-19 from cohort 1 had a history of liver disease, yet LFTs at admission showed elevated AST in 63% and ALT in 39% of patients. **c**,**d**, AST and ALT levels in patients with COVID-19 acquired during hospitalization worsened after COVID-19 diagnosis in 81% and 67% of patients, respectively. **e**, Demographic overview of cohort 2. *n* = 1,219 patients required hospitalization due to COVID-19 (Michigan). Only 2.4% of admitted patients from cohort 2 had a history of liver disease, yet LFTs at admission show elevated AST in 57% and ALT in 37% of patients with COVID-19. **f**, Variation of mean AST and ALT over time in patients with COVID-19, showing elevations in LFTs associated with mortality. Source data

In this study, we examined three patient cohorts to characterize severe acute respiratory syndrome coronavirus 2 (SARS-CoV-2)-associated liver injury and infection. First, a clinical cohort (Hamburg; cohort 1, *n* = 99) was used to examine changes in liver function tests (LFTs) in patients admitted to hospital due to coronavirus disease 2019 (COVID-19) and those who were diagnosed with COVID-19 during hospitalization (Fig. 1a); 42% of these patients were at least 65 years old, 70% were males, only 23% had at least 3 coexisting conditions (Source Data Fig. 1). Among patients admitted due to COVID-19 (*n* = 72), raised aspartate aminotransferase (AST) and alanine aminotransferase (ALT) were shown in 63 and 39% of cases (Fig. 1b), respectively despite the low frequency of liver disease (1.4%), confirming previous studies^4–11^. Next, we analysed a subgroup of patients that were admitted to hospital due to alternative diagnoses (*n* = 27) and suffered from nosocomial SARS-CoV-2 infection. Both AST and ALT were significantly increased after the diagnosis of COVID-19 (Fig. 1c). While the percentage of patients with raised ALT almost doubled before and after COVID-19 diagnosis (33 versus 59%), the percentage of patients with elevated AST almost tripled (22 versus 67%) (Fig. 1d). Next, we evaluated a large validation cohort of patients admitted to hospital due to COVID-19 (Michigan; cohort 2, *n* = 1,219) (Fig. 1e); 44% of patients were at least 65 years old, 57% were males and only 16% had at least 3 coexisting conditions (Supplementary Table 1). Importantly, 57% (*n* = 699) of patients showed elevations of AST and 37% (*n* = 452) of ALT at admission (Fig. 1e). Furthermore, LFT elevations at admission and during the second week of hospitalization were associated with mortality (Fig. 1f), raising questions about their role in disease severity. Together, these observations clearly highlight hepatic injury as an important clinical feature of patients with COVID-19 requiring hospitalization.

**Fig. 2:**
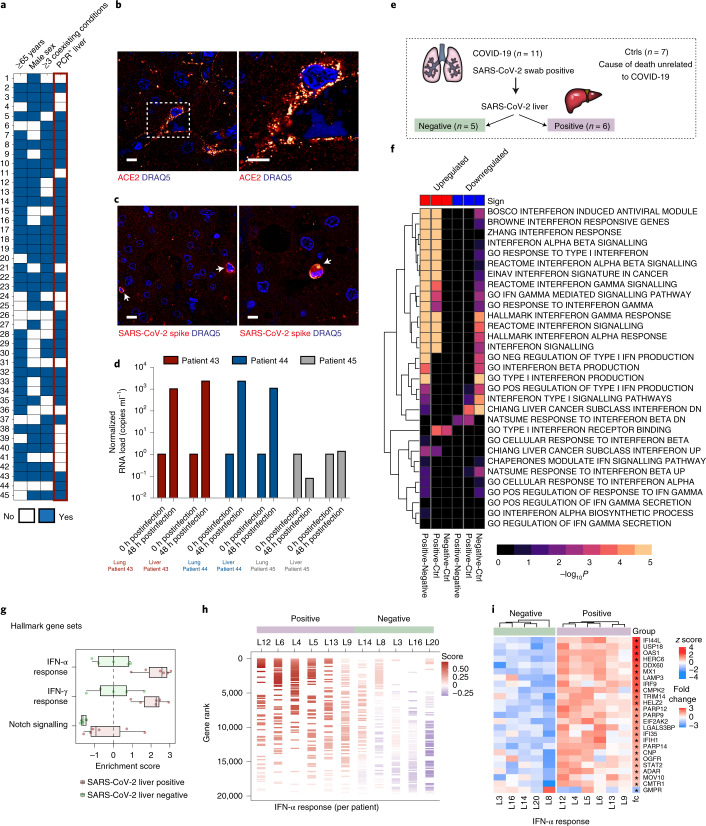
SARS-CoV-2 liver tropism is associated with transcriptional regulation of IFN responses. **a**, Clinical heatmap of 45 patients indicating age ≥65 years, sex, 3 or more coexisting conditions and SARS-CoV-2 liver tropism (PCR + liver). **b**, Immunofluorescence images show the presence of the SARS-CoV-2 receptor ACE2 in hepatic cells (that is, Kupffer cells). Staining was performed in samples from five different patients (data shown in Extended Data Fig. 2). **c**, SARS-CoV-2 spike protein detection in autopsy liver tissues (that is, Kupffer cells and hepatocytes). **d**, Successful SARS-CoV-2 isolation in postmortem livers and respective lungs from two of three autopsy cases, showing increases in SARS-CoV-2 RNA levels in the supernatants of infected cells (43–45 refers to the number sequence in **a**). **e**, Schematic of autopsy tissue selection for molecular profiling. **f**, Generally applicable gene set enrichment (GAGE) analysis of gene sets significantly regulated between liver PCR-positive, PCR-negative and Ctrl conditions. **g**, Single-sample jitter plot for gene sets significant (*P*_adj_ < 0.05) in at least 1 sample and significant difference between the PCR-positive and PCR-negative groups (Wilcoxon signed-rank test, *P* < 0.05). Box plot (box extending from the 25th to the 75th percentile with the median shown as a line in the middle and the whiskers indicating the smallest and largest values) showing the most relevant pathways from Consensus PathDB. Pathways were ranked according to the average difference between PCR-positive and PCR-negative. Each dot represents the enrichment score from the single-sample analysis. Two-sided Wilcoxon signed-rank tests were performed for statistical significance, including IFN-α response (*P* = 0.004329004), interferon-γ (IFN-γ) response (*P* = 0.017316017) and Notch signalling (*P* = 0.017316017). **h**, Single-sample enrichment barcode illustrating the distribution of the hallmark gene set ‘IFN-α response’ in every single sample. Genes were ranked according to their normalized TPM value. The colour coding represents the enrichment score of the gene set during a random walk over the ranked list of genes. Samples were divided into PCR-positive and PCR-negative and then ordered from left to right based on their enrichment score, from high to low. **i**, Row-wise scaled intensity (*z*-score) heatmap showing genes from the IFN-α response gene set. Genes were ranked according to the log_2_ fold change, which is indicated on the right. *P_adj_ < 0.05. Within each group, samples were clustered based on Euclidean distance. Scale bars, 10 μm. Source data

It has been postulated that LFT elevations in hospitalized patients with COVID-19 may result from systemic inflammation or severe cellular stress (for example, hypoxia), as generally observed in critically ill patients^9^. However, an autopsy study reported ultrastructural evidence of SARS-CoV-2 (ref. ^12^), and a second study reported histopathological findings in a liver biopsy of a patient with abnormal liver enzymes, including no obvious inflammation in the portal area, with normal description of the interlobular bile duct, interlobular vein, interlobular artery and hepatocytes with minimal inflammatory cell infiltration^11^. Given that liver biopsies are not routinely performed in patients with COVID-19 with altered LFTs, we evaluated a third cohort (cohort 3, *n* = 45 autopsy cases) in search of direct evidence of liver infection (Supplementary Table 2); 73% (*n* = 33) were older than 65, 69% (*n* = 31) had at least 3 coexisting conditions and 62% (*n* = 28) were males, matching the demographic characteristics linked to severe COVID-19. SARS-CoV-2 RNA was detected using quantitative PCR with reverse transcription (RT–qPCR) targeting the E gene in 69% of cases (*n* = 31) (Fig. 2a), which was more frequently associated with older age, male sex and multiple coexisting conditions, as shown in previous reports^2,13,14^.

**Extended Data Fig. 1:**
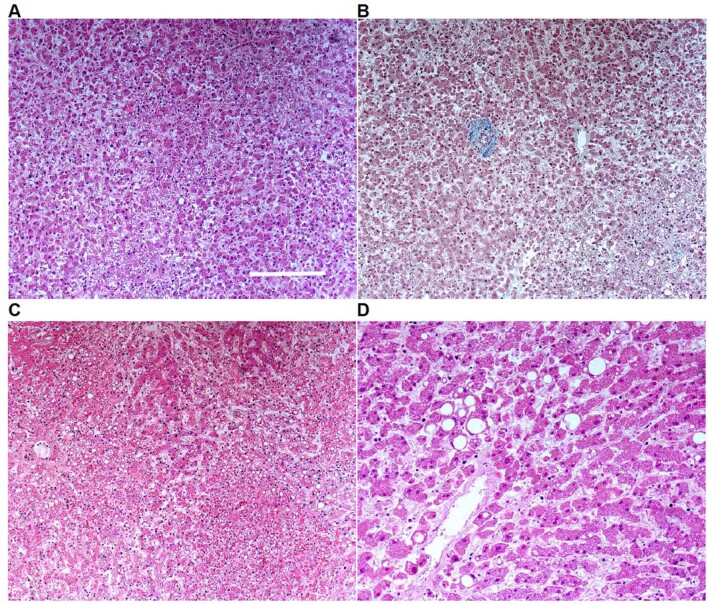
Histopathology in autopsy samples. **a, b**, Histological sections from COVID-19 autopsy tissue without signs of overt cytopathic changes. Examples of other pathological alterations. **c**, Overview of a case with focal fatty changes and centrilobular necrosis, likely as a consequence of shock. **d**, Zoom in of a case with moderate fatty liver and marked centrilobular necrosis, also likely due to shock. These images represent the main findings after careful examination of 18 cases. Scale bar represents 350um.

**Extended Data Fig. 2:**
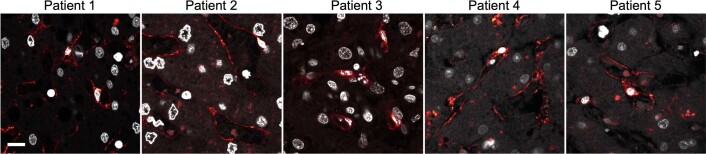
ACE2 expression. ACE expression (orange) and DNA (grey) in the human liver. Based on anatomical location, the staining pattern suggests expression by Kupffer cells. All images were taken at the same magnification. Scale bar represents 10um. This experiment was repeated 3 times in 3 samples with identical results. Source data

**Extended Data Fig. 3:**
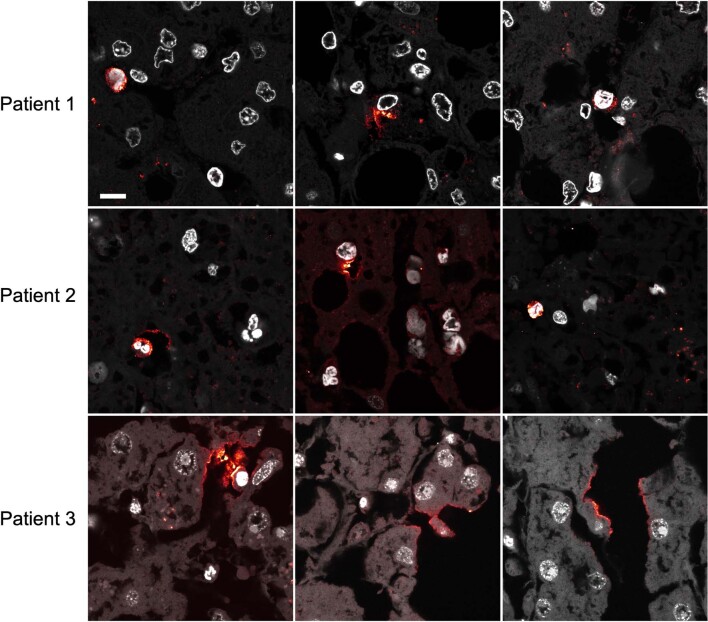
SARS-CoV-2 spike protein in autopsy samples. **a**, Expression of SARS-CoV-2 spike protein (orange) and DNA (grey) in autopsy tissue from 3 patients with confirmed RT-qPCR^+^ for SARS-CoV-2 in the respiratory tract (during clinical course) and in the liver (at autopsy). Based on anatomical locations, these findings suggest expression of SARS-CoV-2 spike protein in Kupffer cells, immune cells, and hepatocytes. Scale bar represents 10um. Source data

Pathology assessment only revealed signs of shock, probably due to severe systemic disease, and adipose changes without classical signs of hepatitis or significant immune infiltration (*n* = 18; Extended Data Fig. 1). However, a combination of indirect immunofluorescence and high-resolution confocal microscopy identified hepatic expression of angiotensin-converting enzyme 2 (ACE2), the main cellular receptor of SARS-CoV-2 (Fig. 2b and Extended Data Fig. 2; *n* = 5 liver samples), and SARS-CoV-2 spike protein (Fig. 2c and Extended Data Fig. 3, *n* = 3 examples). In addition, SARS-CoV-2 RNA was also found in hepatic cells with in situ hybridization (Supplementary Fig. 1; *n* = 4, that is, *n* = 2 SARS-CoV-2 PCR^+^ and *n* = 2 controls (Ctrls)), confirming the spatial location of SARS-CoV-2 in the human liver using multiple techniques that complement the findings of previous studies^12,15^. To provide additional context, we first compared the levels of SARS-CoV-2 RNA copies per cell between airway samples (previously reported in Puelles et al.^2^) and a subset of autopsy livers, showing a similar range but a lower median viral RNA load in hepatic specimens (Supplementary Fig. 2a; *n* = 18 lung, *n* = 16 pharynx and *n* = 18 liver), and we quantified the number of SARS-CoV-2 spike-positive cells in a small subset of our autopsy samples (*n* = 6 liver samples, that is, *n* = 3 SARS-CoV-2 PCR^+^ and *n* = 3 Ctrls with quantification provided in Supplementary Fig. 2b), both of which may explain the lack of overt cytopathic changes (that is, hepatitis). Notably, we isolated infectious SARS-CoV-2 from 2 out of 3 autopsy livers and corresponding lungs, confirming the infectivity of postmortem liver tissue samples, leading to a 1,000 time increase in viral RNA after 48 h of cell infection in vitro (Fig. 2d), as previously reported in the kidney^13^. Together, our data provide spatial and functional evidence of SARS-CoV-2 hepatic tropism, at least in cases of fatal COVID-19.

**Extended Data Fig. 4:**
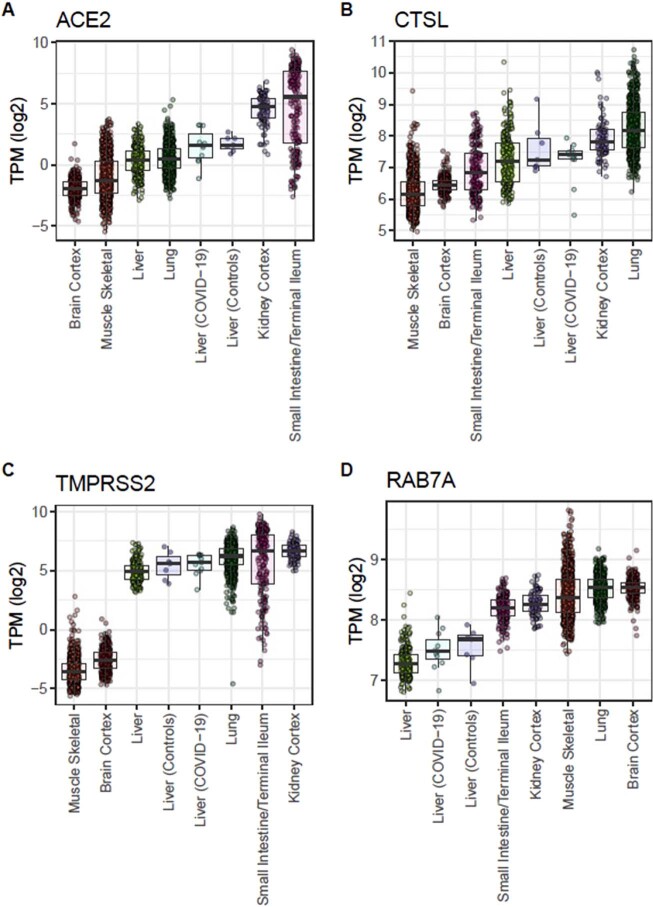
Expression of known SARS-CoV-2 entry receptors and facilitators in liver autopsy samples. **a**, Gene expression of ACE2 in different GTEX tissues shows log2 transcript per million (TPM) expression levels in the liver after intestine, kidney and lung. ACE2 expression levels in the liver samples of COVID-19 patients and controls are comparable with liver and lung expression in GTEX data set. **b**, Gene expression of CTSL and **c**, TMPRSS2 and **d**, RAB7A in different GTEX tissues shows log2 TPM expression levels in the liver after intestine, kidney and lung. Box plots showing boxes from the 25th to the 75th percentile with the median shown as a line in the middle and whiskers indicating 1.5 times the interquartile range.

**Extended Data Fig. 5:**
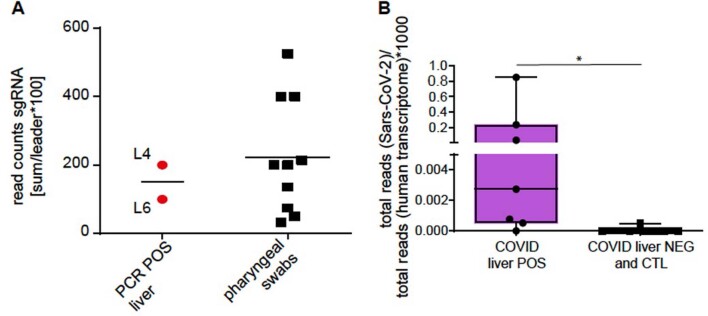
Detection of SARS-CoV-2 subgenomic RNA. **a**, Relative fraction of sgRNA in SARS-CoV-2-positive liver samples is comparable to pharyngeal swab sample data (medRxiv 2020.06.11.20127332; doi: 10.1101/2020.06.11.20127332). **b**, Total number of SARS-CoV-2-positive reads divided by total number of human reads (*1000) shows a higher number of reads aligning to the SARS-CoV-2 genome in SARS-CoV-2 liver POS samples than in SARS-CoV-2 liver NEG samples and controls. *, p-value = 0.0122 (Mann-Whitney test, two-tailed). Box plot: box extending from the 25th to the 75th percentile with the median shown as a line in the middle and whiskers indicating smallest and largest values.

Next, we aimed to determine the molecular changes associated with SARS-CoV-2 liver tropism. Thus, liver tissues from a subset of COVID-19 autopsies were selected for transcriptomic and proteomic analyses (*n* = 11; Fig. 2e). Briefly, 3 comparison groups were defined a priori: (1) patients with COVID-19 and SARS-CoV-2-positive livers (*n* = 5 for transcriptomics and *n* = 6 for proteomics); (2) patients with COVID-19 and SARS-CoV-2-negative livers (*n* = 5 for transcriptomics and proteomics) to account for the systemic effect of COVID-19; and (3) patients with non-COVID-19-related deaths (*n* = 5 for proteomics and *n* = 7 for transcriptomics, that is, Ctrls) to account for pleiotropic effects resulting from autopsy-related collection and storage. The groups were similar in terms of age, sex and number of coexisting conditions (Supplementary Table 3). First, transcriptomic profiling confirmed the expression of known SARS-CoV-2 entry receptors and facilitators in the liver autopsy samples (Extended Data Fig. 4), including ACE2, transmembrane protease serine 2 (TMPRSS2), procathepsin L and Ras-related protein Rab-7a^16,17^. Importantly, a direct comparison to a publicly available dataset^18^ contextualized the expression of these facilitators in relation to other organs, where liver samples showed similar expression levels as the lung, only surpassed by the kidney and small intestine, suggesting that hepatic tissue is susceptible to SARS-CoV-2 infection. Then, we detected SARS-CoV-2 subgenomic RNA within our own dataset and identified two samples out of six with values in the range of the infected respiratory tract, tracing SARS-CoV-2 to the liver by another independent methodology (Extended Data Fig. 5).

**Extended Data Fig. 6:**
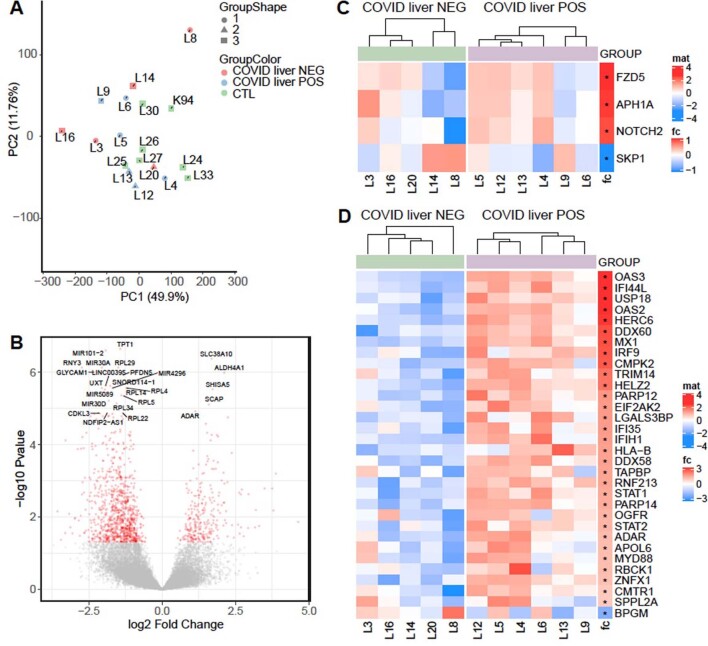
Transcriptional changes associated with SARS-CoV-2 hepatic tropism. **a**, Principal Component Analysis performed on the normalized intensity showing PCR positive (blue), PCR negative (red) Covid-19 liver samples and Control liver (green) samples. PCR- samples are well embedded with healthy liver tissue. **b**, Volcano plot shows the differentially regulated genes (red dots, adjusted p-value < 0.05) in SARS-CoV-2 PCR positive vs. negative liver samples. Top 25 regulated genes, based on p-value, are labeled. **c**, Row-wise scaled intensity (mat, *z*-score) heatmap showing genes from Notch signaling pathway and **d**, Interferon gamma response gene-set. Genes are ranked according to the log2 fold change, indicated on the right side. Adjusted p-value < 0.05 is indicated with ‘*’. Within each group, samples are clustered based on Euclidean distance. Source data

**Extended Data Fig. 7:**
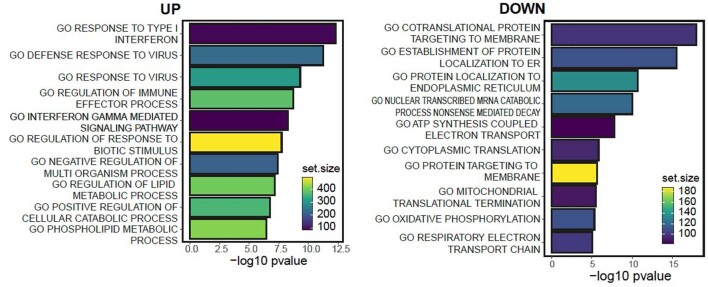
Gene ontology analysis. Barplots showing the top 10 UP- and DOWN-regulated gene-sets from Gene Ontology Biological processes between PCR positive and negative samples. Color code represent the number of genes within each gene set.

Transcriptional changes associated with SARS-CoV-2 hepatic tropism revealed a significant upregulation of type I and II interferon (IFN) responses (Fig. 2f and Extended Data Fig. 6). Gene Ontology (GO) analysis confirmed upregulation of IFN and viral responses and increases in lipid and phospholipid metabolic processes (Extended Data Fig. 7), supporting a recent study linking perturbations of lipid metabolism with SARS-CoV-2 infection^19^. Importantly, these findings were also consistent at a single patient level (Fig. 2g) and within single gene sets (Fig. 2h,i). While SARS-CoV-2 has developed strategies to evade IFN responses, leading to relatively weak type I and III IFN induction in host cells^20^, a recent study showed a high expression level of IFN-stimulated genes in patients with a high SARS-CoV-2 viral load and high levels of pro-inflammatory cytokines but relatively intact lung morphology^21^. These observations are in agreement with our findings, supporting upregulated IFN responses in tissues with traces of SARS-CoV-2 RNA and without classical cytopathic changes. In addition, we also identified that components of IFN-related JAK-STAT signalling (Supplementary Fig. 3), and other central cellular processes involved in lipid/cholesterol synthesis, including terpenoid and steroid biosynthesis were all modulated in patients with SARS-CoV-2 liver tropism. Altogether, these data provide a comprehensive framework to understand the molecular consequences of SARS-CoV-2-mediated liver injury.

**Fig. 3:**
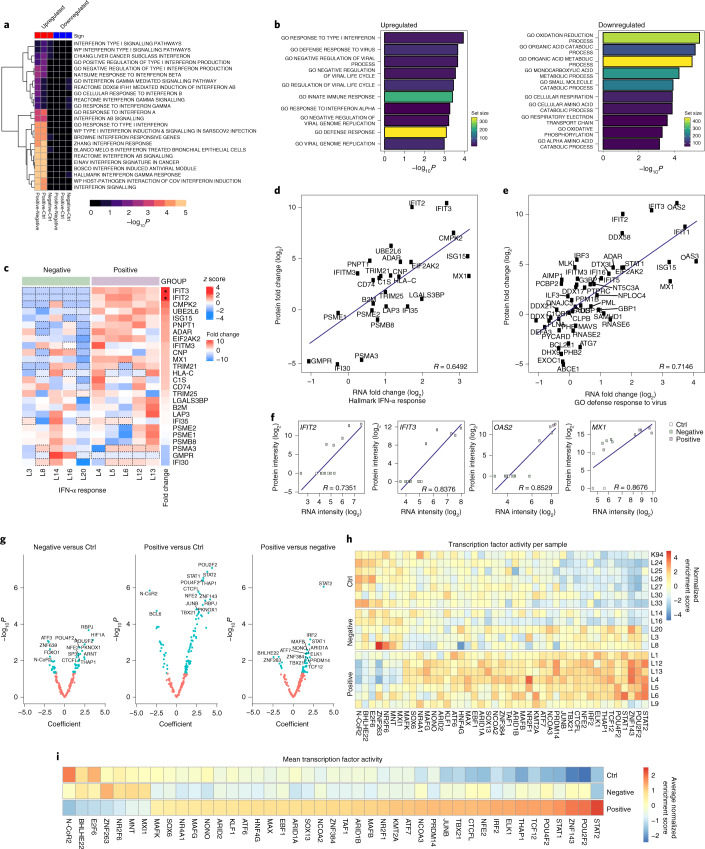
SARS-CoV-2 liver tropism is associated with proteomic and transcription factor activity regulation of IFN responses. **a**, GAGE analysis of gene sets significantly regulated between liver PCR-positive (*n* = 5), PCR-negative (*n*= 5) and Ctrl (*n* = 5) conditions. **b**, Bar plots showing the top 10 up- and downregulated gene sets from GO Biological Processes between positive and negative. The colour coding represents the number of genes within each gene set. **c**, Row-wise scaled intensity (*z*-score) heatmap showing genes from the ‘IFN-α response’ gene set. Genes were ranked according to the log_2_ fold change, which is indicated on the right. *P_adj_ < 0.05. Within each group, samples were clustered based on Euclidean distance. **d**,**e**, mRNA versus protein-positive versus negative fold change scatter plot of gene sets ‘hallmark IFN-α response’ (**d**) and ‘GO defense response to virus’ (**e**). Each dot represents a gene that belongs to the gene set. The blue line represents the linear regression between mRNA and protein fold changes. **f**, mRNA versus protein intensity scatter plot of the genes *IFIT2*, *IFIT3*, *OAS2* and *MX1*. Each dot represents a single sample, colour-coded based on the groups: white, green and purple for Ctrl, negative and positive, respectively. The blue line represents the linear regression between mRNA and protein-normalized intensities. **g**, Transcription factor analysis revealed differential transcription factor usage in Ctrl, COVID-19 liver negative and liver positive samples. **h**, Heatmap of differentially regulated transcription factors per sample (positive: *n* = 7; negative: *n* = 6; Ctrl: *n* = 6). **i**, Summary of the mean transcription factor analysis. Source data

**Extended Data Fig. 8:**
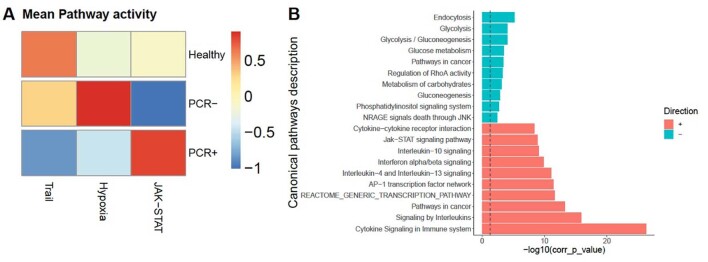
Canonical pathway analysis. **a**, Transcription factor analysis reveals differential transcription factor usage in CTL, COVID-19 liver NEG and liver POS samples. Summary of mean pathway activity in each infection status. **b**, Canonical pathways up (+) or down (-) regulated.

Global proteomic profiling in hepatic tissues of the same autopsy cohort showed substantial regulation of IFN, viral and in particular SARS-CoV-2 responses (Fig. 3a and Source Data Fig. 3). Protein ontology analysis revealed upregulation of IFN signalling and downregulation of basic biological processes, including oxidation reduction, oxidative phosphorylation and cellular respiration (Fig. 3b). Type I IFN responses were confirmed at the single patient level (Fig. 3c). Gene/protein sets for hallmark ‘interferon-α (IFN-α) response’ (*R* = 0.65, *P* < 0.05; Fig. 3d) and the GO Biological Process ‘defence response to virus’ (*R* = 0.71, *P* < 0.05; Fig. 3e), as well as single gene/protein analysis for IFN-inducible genes (*R* = 0.74–0.87, *P* < 0.05; Fig. 3f), showed strong associations between transcriptomic and proteomic profiles, highlighting a coherent modulation of SARS-CoV-2-related pathways at the transcriptional and translational level. Furthermore, transcription factor activity profiling (Fig. 3g) provided individual (Fig. 3h) and global (Fig. 3i) signatures that combined transcription factor upregulation (for example, signal transducer and activator of transcription 2 (STAT2), a signal transducer and activator of transcription that mediates signalling by type I IFNs) and downregulation (that is, nuclear receptor corepressor 2 (N-CoR2), which aids histone deacetylases to modify chromatin structure, thus preventing basal transcriptional activity). Canonical pathway analysis confirmed upregulation of immune-mediated, IFN and JAK-STAT-related responses (Extended Data Fig. 8a), as well as downregulation of glucose and carbohydrate metabolism in association with liver tropism (Extended Data Fig. 8b). Interestingly, modulation of JAK-STAT signalling has been proposed to target SARS-CoV-2 viral entry and replication^22^ in a study that dissected SARS-CoV-2 infectivity using organotypic three-dimensional cultures of primary human liver cells.

**Fig. 4:**
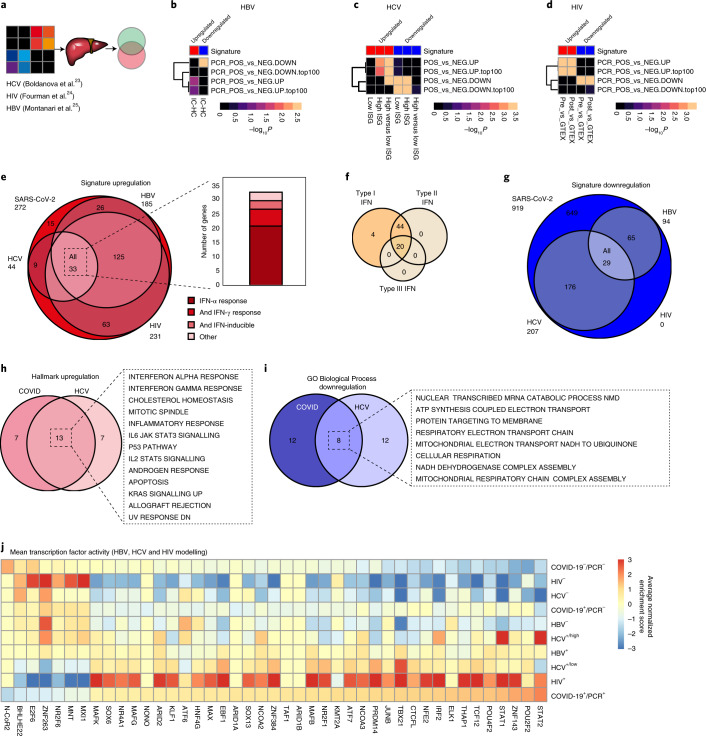
SARS-CoV-2 shares molecular signatures with viruses associated with liver injury. **a**, Publicly available datasets from HBV, HCV and HIV infections were compared with our COVID-19 up- and downregulated gene signatures via GSEA. **b**, HBV infection showed overlapping gene signatures with our data (upregulated genes/top 100 upregulated genes). **c**, Liver biopsies from HCV-positive patients showed overlapping gene signatures with our data in high IFN-stimulated genes (ISG) patients and high versus low ISG patients (upregulated genes/top 100 upregulated genes) or high and low ISG (downregulated genes/top 100 downregulated genes). **d**, HIV infection combined with liver steatosis showed a larger overlap with our COVID-19 signature. **e**, Overlapping genes with the leading edge of the GSEA of the HBV, HCV and HIV datasets with our upregulated gene signatures showing most genes overlapping all four datasets belonging to the gene sets, IFN-α and IFN-γ responses or having been described as IFN-inducible. **f**, Overlapping genes from **e** were classified using www.interferome.org (v2.01) as type I or type II IFN-specific. **g**, Overlapping genes with the leading edge of the GSEA of the HBV, HCV and HIV datasets with our downregulated gene signatures showing a small overlap with no significant gene signature. **h**, Hallmark pathways upregulated for COVID-19 liver positive versus negative samples and HCV ISG high versus Ctrl showing overlap in 13 out of 20 pathways. **i**, GO Biological Process downregulated for COVID-19 liver positive versus negative samples and HCV ISG high versus Ctrl showing overlap in 8 out of 20 pathways. **j**, Mean transcription factor activity showed similar transcription factor use in COVID-19 liver positive samples, HIV-positive and HCV low ISG, followed by HBV-positive and HCV high ISG samples.

The clinical and molecular data from our study suggest that SARS-CoV-2-mediated liver injury is comparable to previously characterized hepatotropic viruses. For this reason, we compared publicly available datasets containing bulk RNA sequencing (RNA-seq) data from liver tissue samples from patients with hepatitis C virus (HCV)^23^, human immunodeficiency virus (HIV)^24^ and hepatitis B virus (HBV)^25^ via gene set enrichment analysis (GSEA) (Fig. 4a). While our literature search was carefully performed, it was not possible to find studies that could be perfectly matched our study demographics (for example, our population was notably older than the other three studies as per the demographic trends of fatal COVID-19; Supplementary Table 4). Interestingly, and despite of multiple methodological limitations, all three infections affecting the liver, including HBV (Fig. 4b), HCV (Fig. 4c) and HIV (Fig. 4d), showed overlapping gene expression patterns with our proposed SARS-CoV-2 signature. Specific examples include upregulation of IFN signalling gene sets (Fig. 4e), especially types I, II and III IFN responses (Fig. 4f), and downregulation of viral gene expression and related cellular functions (that is, translational initiation and peptide metabolic processes) (Fig. 4g). Pathway analysis also revealed remarkable overlap between SARS-CoV-2 and HCV (13 of 20 pathways for upregulated; Fig. 4h; 8 of 20 pathways for downregulated; Fig. 4i). In addition, we identified similar transcription factor use in livers from patients with severe COVID-19 and patients with liver steatosis and HIV, inactive carriers of HBV and chronic HCV (Fig. 4j). Taken together, this comparative analysis highlights a shared molecular signature between SARS-CoV-2 and multiple viruses with well-characterized patterns of liver injury.

**Extended Data Fig. 9:**
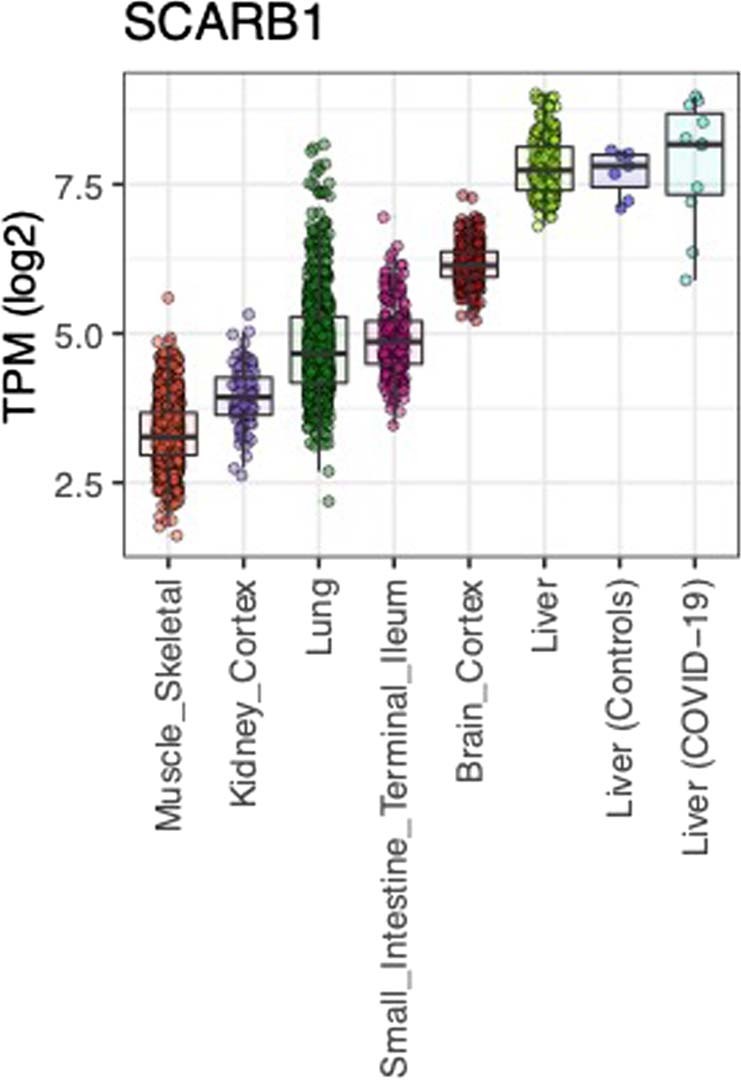
Gene expression of SCARB1. Gene expression of SCARB1 in different GTEX tissues shows log2 transcript per million (TPM). Our own datasets are also used to provide context, including Liver (Controls) and Liver (COVID-19). Box plots showing boxes from the 25th to the 75th percentile with the median shown as a line in the middle and whiskers indicating 1.5 times the interquartile range.

**Extended Data Fig. 10:**
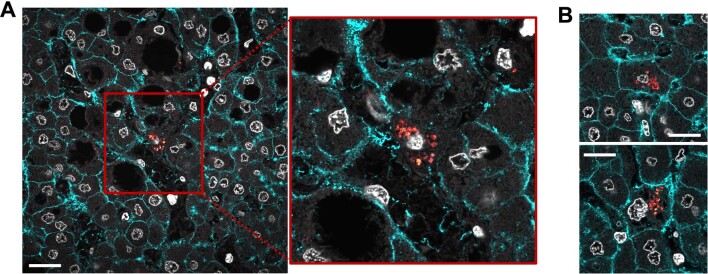
Protein expression of SR-B1 and SARs-CoV-2 spike in autopsy tissue. Protein expression of SR-B1 (cyan) in one post-mortem liver sample – and co-expression with SARS-CoV-2 spike protein (orange) within the same cell. In A, we show an overview of a large liver region, showcasing widespread expression of SR-B1 among hepatocytes with a zoom-in to a cell expressing also SARS-CoV-2 spike. In B, we show two additional regions with hepatocytes showing co-expression of SR-B1 and SARS-CoV-2 spike in the same cell. All images were performed in one specimen as proof-of-principle. Scale bars represent 20um. Source data

Since the topic of SARS-CoV-2 hepatic tropism has been highly debated^3^, we aimed to tackle this question from multiple perspectives, including clinical (two large independent patient cohorts that showcase COVID-19-associated liver injury), histopathological (identification of SARS-CoV-2 at the RNA and protein levels with high spatial resolution), virological (RT–qPCR in human autopsy samples and isolation of SARS-CoV-2 from human autopsy liver specimens), molecular (transcriptomic, proteomic and transcription factor activity use profiling, indicating the presence of SARS-CoV-2 in association with significant tissue responses) and bioinformatic (detection of subgenomic RNA and data validation with external public datasets of virus-mediated hepatic injury). Despite all these layers of evidence supporting SARS-CoV-2 hepatic tropism, the exact mechanism of infection is unclear. Our findings show that the human liver is susceptible to SARS-CoV-2 infection since cell entry receptors and facilitators are clearly expressed in hepatic cells. However, we observed a mismatch between the expression of the ACE2 protein, mostly observed in Kupffer cells, and the location of the SARS-CoV-2 spike protein, which was observed in Kupffer and parenchymal cells (for example, hepatocytes). In this context, it is worth considering a recent study that proposed high-density lipoprotein scavenger receptor class B member 1 (SRB1) as a cell entry facilitator for SARS-CoV-2. SRB1 acts as a critical receptor that affects HCV entry^26^, an infection that shares some molecular features with SARS-CoV-2 liver tropism. While Wei et al.^19^ reported strong protein expression of SRB1 in the human liver, we confirmed messenger RNA expression of *SCARB1* (SRB1 gene) in multiple human organs, showing the highest expression levels in the liver followed by the brain, small intestine, lung, kidney and muscle (Extended Data Fig. 9). In addition, we confirmed SRB1 protein expression on human hepatocytes and in association with the expression of SARS-CoV-2 spike protein (Extended Data Fig. 10). It is plausible that even if the expression of ACE2 in hepatocytes is relatively low at both RNA and protein levels, high levels of SRB1 may enhance the potential for SARS-CoV-2 entry.

It is worth remembering that our observations represent a late ‘snapshot’ of a severe disease state and consequent multi-organ dysfunction (that is, severe COVID-19 leading to death), which provides a limited view affected by disease course, coexisting conditions, therapeutic interventions and postmortem processes. All these factors may influence our view of receptor expression patterns (for example, potential up/downregulation during infection), definition of cell-specific tropism (that is, severe injury to infected cells and local protein clearance) and even molecular signatures, which likely reflect profound cellular changes in neighbouring cells that have survived an active phase of SARS-CoV-2 tropism and could explain the common features between patients with severe COVID-19 and hepatotropic viruses (that is, HCV). Furthermore, our findings should be carefully considered in the context of lack of access to full clinical data in our large clinical cohorts, limiting our ability to rule out other causes of liver injury (for example, drug-mediated hepatoxicity), and relatively small sample sizes in our multi-omics experiments, which were mainly focused on patients who died due to severe COVID-19, raising questions about generalizability to mild and moderate cases. Despite this, a recent clinical study followed 443 non-hospitalized individuals for approximately 9.6 months after their first positive SARS-CoV-2 test, revealing signs of multi-organ injury^27^, which allows us to question the potential for similar molecular changes in mild and moderate forms of COVID-19.

In summary, our findings provide multimodal evidence of SARS-CoV-2 human liver tropism and a molecular signature, featuring a strong upregulation of IFN responses, JAK-STAT signalling and liver-specific metabolic modulation. This unique human dataset can be used as a rational framework for future studies aiming to characterize the potential consequences of SARS-CoV-2 extrapulmonary manifestations and identify personalized therapeutic strategies.

## Methods

### Tissue and data collection

Autopsies were performed at the Institute of Legal Medicine of the University Medical Center Hamburg-Eppendorf. From every liver specimen collected by the Institute of Legal Medicine, multiple randomly chosen small samples were available for different analyses. The ethics committee of the Hamburg Chamber of Physicians was informed about the study (nos. 2020-10353-BO-ff and PV7311). Ctrls included cases of sudden, non-infectious deaths. The postmortem interval was on average 6 d. Informed consent was obtained from a next of kin or legal representative for autopsy and tissue sampling. No compensation was paid.

The study protocol for clinical data collection (patient cohorts) was approved by the institutional review board (IRB) of the University of Michigan (no. HUM00178971) and Hamburg (no. WF-052/20). The IRB approved a waiver of informed consent for this observational study.

### Molecular detection of SARS-CoV-2

Tissue samples were systematically sampled during the autopsy procedure in 45 individuals. Automated nucleic acid extraction was performed according to the manufacturer’s recommendations with whole process control (control kit; Roche), with a final elution volume of 100 μl. For virus quantification, a previously published assay was adopted with modifications using chimeric 2′-O-methyl RNA bases at the penultimate base of both primers (mG and mC) to reduce primer dimer formation^28^. The forward primer 5′-ACAGGTACGTTAATAGTTAATAGCmGT-3′ (400 nM end concentration), 5′-TATTGCAGCAGTACGCACAmCA-3′ (400 nM end concentration) and probe 5′-Fam-ACACTAGCC/ZEN/ATCCTTACTGCGCTTCG-Iowa Black FQ-3′ (100 nM end concentration) were used. Primer and probes were obtained from Integrated DNA Technologies. One-step RT–PCR (25 μl volume) was run on the LightCycler 480 system (Roche) using the one-step RNA control kit as master mix (Roche) and 5 μl of eluate. The Ct value for the target SARS-CoV-2 RNA (FAM) was determined using the second derivative maximum method. To quantify the standard in vitro-transcribed RNA, the E gene of SARS-CoV-2 was used. The standard was obtained via the European Virus Archive^4^. The linear range of the assay was between 1 × 10^3^ and 1 × 10^9^ copies ml^−1^. β-Globin qPCR was performed with the commercial TaqMan primer set (catalogue no. 401846; Thermo Fisher Scientific) and Roche DNA control kit. The PCR was run on the LightCycler 480 system. The amount of DNA was normalized using a human DNA standard (KR0454). SARS-CoV-2 RNA levels in tissues were normalized to β-globin DNA.

### Histology, immunolabelling and quantification

Liver tissue was fixed in formaldehyde. Then, 2-μm slides were cut and haematoxylin and eosin, periodic acid–Schiff and Masson–Goldner stainings were performed. Expert pathology review was conducted by two independent and experienced pathologists.

ACE2 and SARS-CoV-2 spike were detected in formalin-fixed paraffin-embedded sections using a protocol for indirect immunofluorescence and confocal microscopy^29,30^. We used primary antibodies against ACE2 (dilution: 1:200, catalogue no. AF933; R&D Systems) and SARS-CoV/SARS-CoV-2 (COVID-19) (dilution 1:200, catalogue no. GTX632604; GeneTex), which were validated in previous studies^2^^,13^. We also used a primary antibody against SRB1 (dilution 1:200, catalogue no. ab217318; Abcam).

For quantification, and considering the size and degree of autolysis, we performed targeted sampling of five fields of view based on the presence of at least one SARS-CoV-2 spike^+^ cell with a random location within the field; for Ctrls, five random fields were chosen from non-autolytic sites. Then, we quantified the number of SARS-CoV-2 spike^+^ cells and the total number of complete nuclei per field, which allowed us to calculate a percentage of positive cells per field. Statistics were performed with Prism v.9.2.0 (GraphPad Software).

### RNA in situ hybridization (RNAscope)

In situ hybridization was carried out to detect virus RNA of SARS-CoV-2 on paraffin sections utilizing the RNAscope 2.5 HD detection kit (catalogue no. 322310; Advanced Cell Diagnostics) according to the manufacturer’s instructions^31^. Briefly, tissue sections were deparaffinized in xylene followed by target retrieval at 95 °C for 10 min. Subsequently, internal peroxidase activity was quenched by hydrogen peroxide incubation for 10 min followed by permeabilization using protease plus treatment at 40 °C for 30 min. The SARS-CoV-2-specific RNAscope probe V-nCoV2019-S (catalogue no. 848561) was hybridized at 40 °C for 2 h. RNAscope probes specific for either the human ubiquitin C mRNA (catalogue no. 310041) or the bacterial dihydrodipicolinate reductase mRNA (catalogue no. 310043) were used as positive or negative Ctrl, respectively. The RNAscope signal was developed with 3,3′-diaminobenzidine and nuclei were counterstained with haematoxylin.

### Cell culture and virus isolation

Liver tissues were homogenized^32^ and 250 μl of the homogenized tissue solution were used to infect Vero cells (CRL-1586; ATCC). Growth was confirmed by RT–qPCR of cell culture supernatants^28^.

### RNA-seq

Sample selection was based on collection date and RNA quality. RNA was extracted with the QIAGEN RNeasy Micro Kit according to the manufacturer’s instructions followed by Agilent Bioanalyzer sample quality control, library preparation with Lexogen CORALL Total RNA and ribosomal RNA depletion. Single-end RNA-seq was done using 75-base pair NextSeq v.2.5 with >30 million reads. Raw reads were trimmed with Trim Galore! v.0.4.3 (http://www.bioinformatics.babraham.ac.uk/projects/trim_galore/) and aligned with RNA STAR: Galaxy Version 2.7.2b^33^. Mapped reads were counted with HTSeq: Galaxy Version 0.9.1.^34^.

#### GTEX data

Raw counts from several healthy tissues were downloaded directly from the GTEX portal (www.gtexportal.org) on 4 March 2020^35^.

#### Differential analysis

Genes with a non-null count in at least two samples were selected. Raw counts were processed with the limma R package v.3.42.2 (ref. ^36^) and differentially regulated genes between PCR-positive and PCR-negative groups were identified after library size normalization. Both groups were compared to GTEX liver. A Benjamini–Hochberg-adjusted *P* < 0.05 was considered significant.

#### Group-wise GSEA

GSEA was performed using the GAGE R package v.2.36.0 (ref. ^37^) with the Molecular Signatures Database (MSigDB) gene set v.7.1, comparing PCR-positive to PCR-negative groups. Both groups were also compared with GTEX liver. A Benjamini–Hochberg-adjusted *P* *<* 0.05 was considered significant.

#### Single-sample GSEA

First, each sample was normalized to healthy liver tissue to depreciate the background noise and emphasize the true signal. Therefore, the average GTEX liver transcripts per kilobase million (TPM) intensity was subtracted for every single gene. In each sample, genes were ranked according to their normalized TPM value and used as input for single-sample enrichment analysis using the fgsea R package v.1.12.0 (ref. ^38^) with MSigDB gene set v.7.1 (ref. ^39^) and ConsensusPathDB v.34 (ref. ^40^). In all gene sets, the enrichment score was calculated based on a random walk over the ranked list of genes. The significance of the enrichment scores was assessed with 1,000 permutations. A Benjamini–Hochberg-adjusted *P* *<* 0.05 was considered significant.

Kyoto Encyclopedia of Genes and Genomes pathways were created with PathView v.1.34.0 (ref. ^41^) with log_2_ positive versus negative fold change.

### Detection of sgRNA

The relative fraction of single-guide RNAs (sgRNAs) was determined by identifying the leader sgRNA fusion containing sequencing reads normalized to read counts containing the genomic RNA leader sequence^42^. Pharyngeal swab data served as a positive control for the detection of sgRNA due to the high amount of replicating virus in this specimen. Swab sample data were reproduced from our own data^42^.

### Proteomic analysis

For sample preparation, paraffin-embedded liver samples were deparaffinized, resuspended in 100 μl lysis buffer (50% trifluoroethanol (TFE), 300 mM Tris-HCl, pH 8.0) and processed^43^. Briefly, samples were sonicated at 4 °C for 15 min (‘high’ settings on a Bioruptor; Diagenode), boiled at 95 °C for 90 min and sonicated again as described above. Cleared lysates were reduced/alkylated (5 mM dithiothreitol, 25 mM chloroacetamide) for 1 h in the dark and concentrated on a vacuum centrifuge (45 min, 60 °C). A total of 50 µg proteins were digested overnight in digestion buffer (10% TFE containing trypsin/LysC (1:50 protein/protein ratio). Digestion was stopped with 1% trifluoroacetic acid (TFA) and peptides were purified on stage tips with two SDB-RPS Empore filter discs (3M) and resuspended in 2% acetonitrile (ACN)/0.1% TFA to a final concentration of 250 ng µl^−1^.

#### Ultra-high-performance liquid chromatography and trapped ion mobility spectrometry quadrupole time of flight settings

Samples were analysed on a nanoElute (plug-in v.1.1.0.27; Bruker) coupled to a trapped ion mobility spectrometry quadrupole time of flight (timsTOF Pro) (Bruker) equipped with a CaptiveSpray source. Peptides (500 ng) were injected into a Trap cartridge (5 mm × 300 μm, 5 μm C18; Thermo Fisher Scientific) and next separated on a 25 cm × 75 μm analytical column, 1.6 μm C18 beads with a packed emitter tip (IonOpticks). The column temperature was maintained at 50 °C using an integrated column oven (Sonation GmbH). The column was equilibrated using 4 column volumes before loading samples in 100% buffer A (99.9% Milli-Q water, 0.1% formic acid (FA)). Samples were separated at 400 nl min^−1^ using a linear gradient from 2 to 17% buffer B (99.9% ACN, 0.1% FA) over 60 min before ramping up to 25% (30 min), 37% (10 min) and 95% of buffer B (10 min) and sustained for 10 min (total separation method time, 120 min). The timsTOF Pro was operated in parallel accumulation-serial fragmentation (PASEF) mode using Compass Hystar v.5.0.36.0. Settings were as follows: mass range 100–1700 *m/z*, 1/K0 start 0.6 V⋅s/cm^2^End 1.6 V⋅s/cm^2^; ramp time 110.1 ms; lock duty cycle to 100%; capillary voltage 1,600 V; dry gas 3 l min^−1^; dry temperature 180 °C. The PASEF settings were: 10 tandem mass spectrometry (MS) scans (total cycle time, 1.27 s); charge range 0–5; active exclusion for 0.4 min; scheduling target intensity 10,000; intensity threshold 2,500; collision-induced dissociation energy 42 eV.

#### Raw data processing and analysis

Raw MS data were processed with the MaxQuant software v.1.6.17 using the built-in Andromeda search engine to search against the human proteome (UniprotKB, release 2019_10) containing forward and reverse sequences concatenated with the SARS-CoV-2 polyprotein with the individual viral open reading frames manually annotated, and the label-free quantitation algorithm^44^. Additionally, the intensity-based absolute quantification (iBAQ) algorithm and match between runs option were used. In MaxQuant, carbamidomethylation was set as fixed and methionine oxidation and N-acetylation as variable modifications. Search peptide tolerance was set at 70 p.p.m. and the main search was set at 30 p.p.m. (other settings left as default). Experiment type was set as TIMS-DDA with no modification to the default settings. Search results were filtered with a false discovery rate of 0.01 for peptide and protein identification. The Perseus software v.1.6.10.4 was used to process the data further. Protein tables were filtered to eliminate the identifications from the reverse database and common contaminants. When analysing the MS data, only proteins identified on the basis of at least one peptide and a minimum of three quantitation events in at least one experimental group were considered. The iBAQ protein intensity values were normalized against the median intensity of each sample (using only peptides with recorded intensity values across all samples and biological replicates) and log-transformed; missing values were filled by imputation with random numbers drawn from a normal distribution calculated for each sample^45^. Differential analysis and GSEA were performed as described above on mRNA.

### Data integration

The mRNA and protein datasets were integrated and the correlation analysis was performed based on two different approaches: gene-wise and sample-wise.

#### Gene-wise

Spearman’s correlation between mRNA and protein across all samples was calculated for every single gene separately. For each gene, lower- and upper-tail *P* values were calculated from the empirical cumulative distribution function (ECDF). Significantly highly correlated genes (upper-tail *P* < 0.05) were selected to perform GSEA using Fisher’s exact test on the MSigDB. A Benjamini–Hochberg-adjusted *P* < 0.05 was considered significant.

#### Sample-wise

For every single gene, we calculated the average fold change between positive and negative. The gene set-specific correlation was assessed by first selecting the genes that belonged to a given gene set, then by calculating the mRNA fold change versus the protein fold change Spearman’s correlation of these genes. To estimate the significance of the correlation for one gene set, correlation values were also calculated from 10,000 random sets of genes, using the same number of genes as the given gene set. Finally, the ECDF was used to return lower- and upper-tail *P* values.

### Transcription factor analysis

Functional analysis was performed with the DoRothEA package (1.4.1)^46^ for transcription factor activity and the PROGENy package (1.14.0)^47^ for pathway activity. To test the difference in activities, a factorial experiment was designed for each modality using the limma package. In both functional types, the first factor was the infection status of the patient (COVID-19^+^/COVID-19^−^) and the second was if there were traces of the virus in their liver (PCR^+^/PCR^−^). Thus, in total there were three factor combinations: a healthy patient (COVID-19^−^/PCR^−^: Ctrl); an early-stage infection (COVID-19^+^/PCR^−^: negative); and a late-stage infection (COVID-19^+^/PCR^+^: positive). A linear model was fitted for each transcription factor and pathway activity to obtain coefficients for each factorial combination. Then, these coefficients were used to compute three contrasts: effect of COVID-19^+^/PCR^−^ based on COVID-19^−^/PCR^−^ samples (negative versus Ctrl); effects of COVID-19^+^/PCR− based on COVID-19^−^/PCR^−^ samples (positive versus Ctrl); and the difference between these two comparisons (positive versus negative). The first and second contrasts were aimed at detecting systematic changes in activity when an early or late-stage COVID-19 infection was taking place. However, the third contrast was aimed at detecting the specific difference in activity between late-stage and early-stage infection. The sign of the obtained coefficients can be interpreted as an increase or decrease of the mean activity for a given comparison, each with an associated probability.

### Reporting Summary

Further information on research design is available in the Nature Research Reporting Summary linked to this article.

## Supplementary information

Supplementary Information Supplementary Figs. 1–3.

Reporting Summary

Supplementary Tables Supplementary Tables 1–4.

## Data Availability

The RNA-seq raw and processed data have been deposited at the University Hamburg Research Data Repository (https://www.fdr.uni-hamburg.de/) with the following identifiers: 10.25592/uhhfdm.8358 and 10.25592/uhhfdm.8372. The mass spectrometry-based proteomics data have been deposited at the ProteomeXchange Consortium (http://proteomecentral.proteomexchange.org) via the PRIDE partner repository with the following dataset identifier: PXD022789. Source data are provided with this paper.

## Source data

Source Data Fig. 1 Demographic data for cohort 1 (Hamburg).
Source Data Fig. 2 ACE2 and SARS-CoV-2 spike microscopy images.
Source Data Fig. 3 Extended proteomic data.
Source Data Extended Data Fig. 2 ACE2 microscopy images.
Source Data Extended Data Fig. 3 SARS-CoV-2 spike microscopy images.
Source Data Extended Data Fig. 6 Extended transcriptomic data.
Source Data Extended Data Fig. 10 SRB1 and SARS-CoV-2 spike microscopy images.
