# Supplementary material for: Molecular consequences of SARS-CoV-2 liver tropism
Source: Nat Metab. 2022 Mar 28;4(3):310–9. doi: 10.1038/s42255-022-00552-6 (PMC8964418; doi:10.1038/s42255-022-00552-6)
Supplement: Supplementary file 1 — Supplementary Figs. 1–3. [file 42255_2022_552_MOESM1_ESM.pdf]

nature portfolio

https://doi.org/10.1038/s42255-022-00552-6

Supplementary information

Molecular consequences of SARS-CoV-2 liver tropism

In the format provided by the authors and unedited

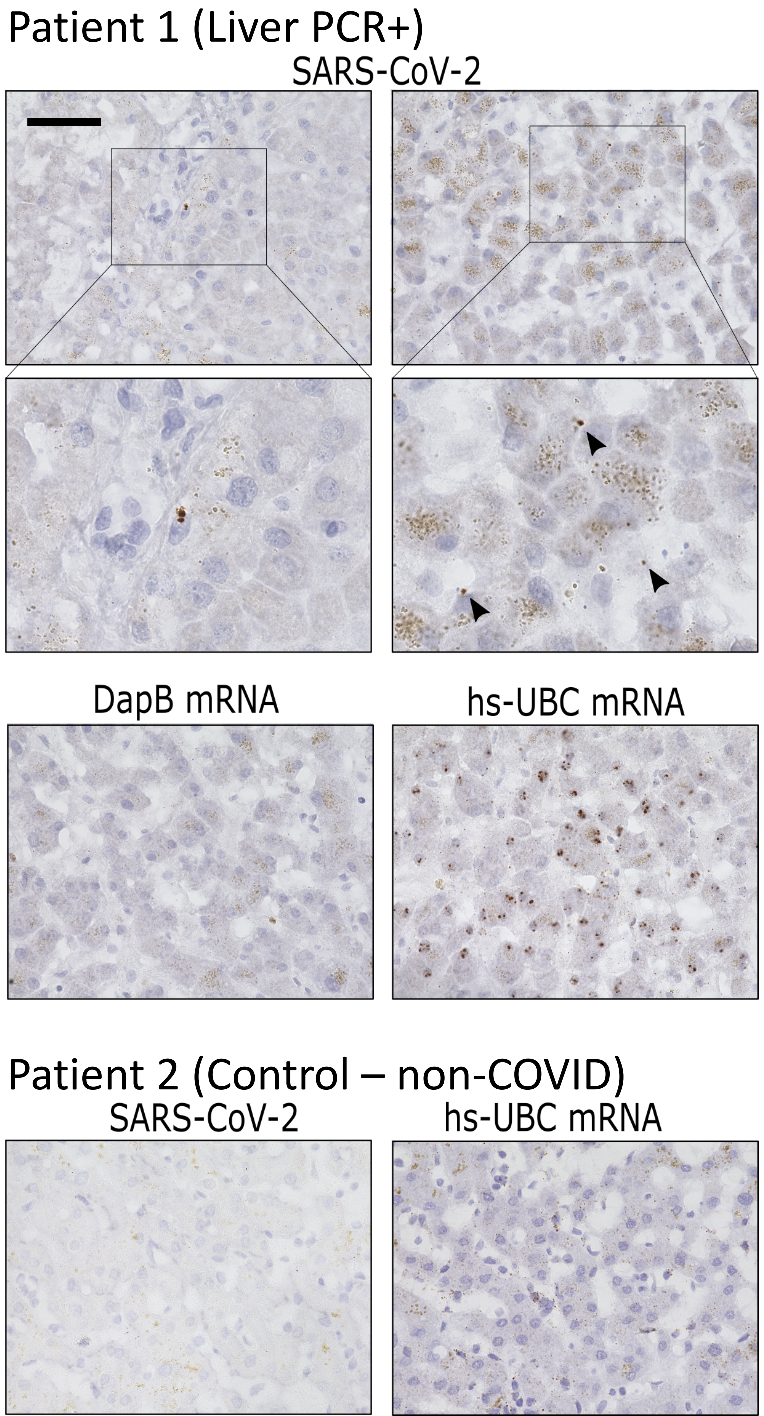

Figure S1:
In situ hybridization detected virus RNA of SARS-CoV-2 in liver tissue samples positive for SARS-CoV-2. RNA integrity was controlled with probes detecting mRNA of the negative and positive control genes DapB and UBC, respectively. Lipofuscin is seen as brownish cellular inclusions in the negative controls. Negative samples for SARS-CoV-2 were probed for SARS-CoV-2 (+)strand RNA, showing no SARS-CoV-2 signal. These images represent two independent biological replicates per group. Scale bar represents 50um.

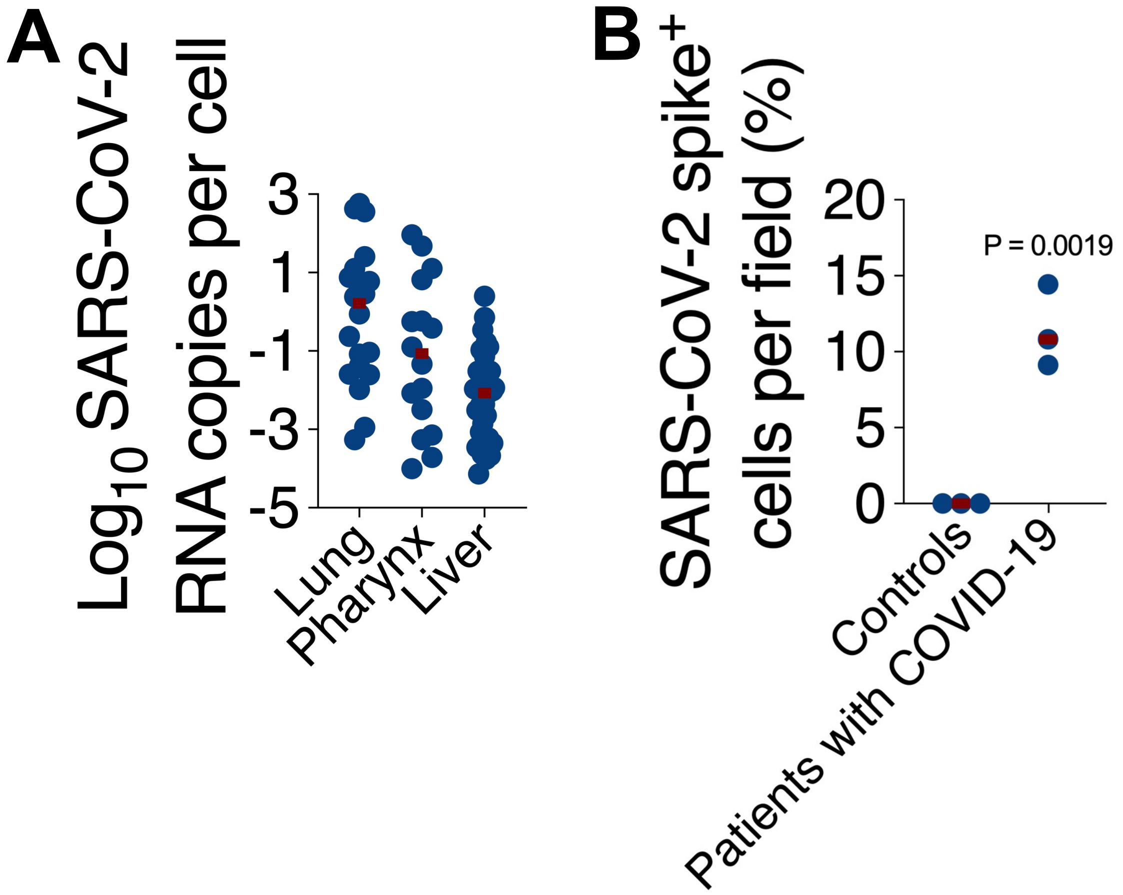

Figure S2:
A, Levels of SARS-CoV-2 copies per cell in autopsy specimens from the respiratory tract (lung and pharynx) and livers. Each dot represents one specimen, red lines represent medians.
B, Quantification of the percentage of SARS-CoV-2 spike positive cells per field of view. A total of 5 field of views (approx. 78um^2) were used per sample in a total of n=3 controls and n=3 patients with COVID-19 and liver tropism. P value was calculated using a two-sided unpaired test.

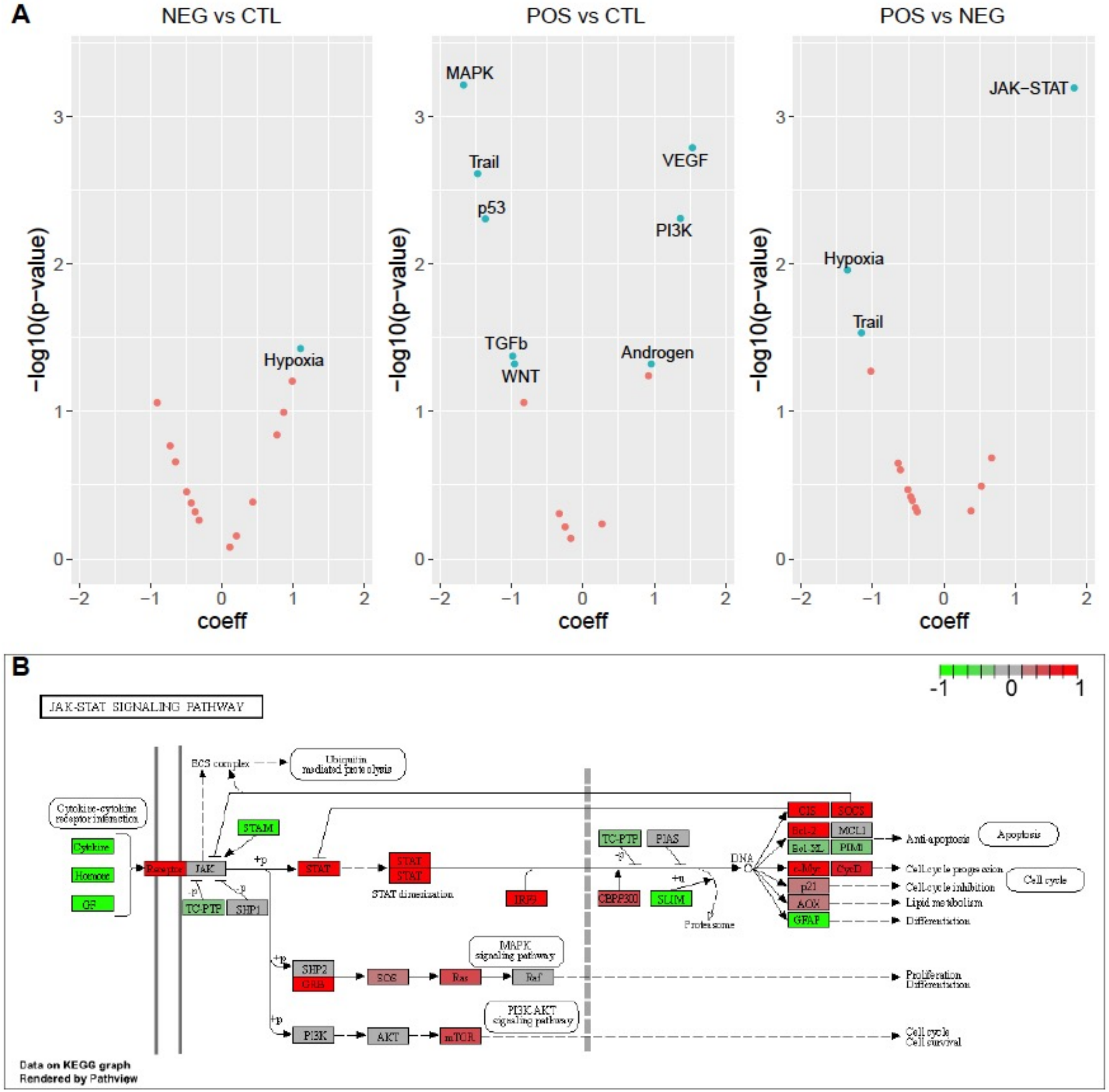

Figure S3:

A, Pathway activity in each infection status shows upregulation of JAK-STAT Signalling pathway in COVID-liver POS samples. B, KEGG pathway „JAK-STAT signalling pathway“ shows log2 foldchange of genes in COVID-19 liver POS vs NEG samples.
