# Supplementary material for: Molecular consequences of SARS-CoV-2 liver tropism
Source: Nat Metab. 2022 Mar 28;4(3):310–9. doi: 10.1038/s42255-022-00552-6 (PMC8964418; doi:10.1038/s42255-022-00552-6)
Supplement: Supplementary file 2 — Reporting Summary [file 42255_2022_552_MOESM2_ESM.pdf]

nature portfolio

Corresponding author(s): Dr. Tobias B. Huber
Dr. Pietro Scaturro
Dr. Victor G. Puelles

Last updated by author(s): Jan 28, 2022

Reporting Summary

Nature Portfolio wishes to improve the reproducibility of the work that we publish. This form provides structure for consistency and transparency in reporting. For further information on Nature Portfolio policies, see our Editorial Policies and the Editorial Policy Checklist.

Statistics

For all statistical analyses, confirm that the following items are present in the figure legend, table legend, main text, or Methods section.

n/a	Confirmed	
☐	☒	The exact sample size (n) for each experimental group/condition, given as a discrete number and unit of measurement
☐	☒	A statement on whether measurements were taken from distinct samples or whether the same sample was measured repeatedly
☐	☒	The statistical test(s) used AND whether they are one- or two-sided<br>*Only common tests should be described solely by name; describe more complex techniques in the Methods section.*
☒	☐	A description of all covariates tested
☐	☒	A description of any assumptions or corrections, such as tests of normality and adjustment for multiple comparisons
☐	☒	A full description of the statistical parameters including central tendency (e.g. means) or other basic estimates (e.g. regression coefficient) AND variation (e.g. standard deviation) or associated estimates of uncertainty (e.g. confidence intervals)
☐	☒	For null hypothesis testing, the test statistic (e.g. F, t, r) with confidence intervals, effect sizes, degrees of freedom and P value noted<br>*Give P values as exact values whenever suitable.*
☒	☐	For Bayesian analysis, information on the choice of priors and Markov chain Monte Carlo settings
☒	☐	For hierarchical and complex designs, identification of the appropriate level for tests and full reporting of outcomes
☒	☐	Estimates of effect sizes (e.g. Cohen's d, Pearson's r), indicating how they were calculated

Our web collection on statistics for biologists contains articles on many of the points above.

Software and code

Policy information about availability of computer code

Data collection	all softwares used to perform data collection are described in the method section of the manuscript.
Data analysis	limma R package v3.42.2, GAGE R package v2.36.0, fgsea R package v1.12.0, PathView, MaxQuant 1.6.17 , DoRothEA, PROGENy, GraphPad Prism v9.2.0

For manuscripts utilizing custom algorithms or software that are central to the research but not yet described in published literature, software must be made available to editors and reviewers. We strongly encourage code deposition in a community repository (e.g. GitHub). See the Nature Portfolio guidelines for submitting code & software for further information.

Data

Policy information about availability of data

All manuscripts must include a data availability statement. This statement should provide the following information, where applicable:
- Accession codes, unique identifiers, or web links for publicly available datasets
- A description of any restrictions on data availability
- For clinical datasets or third party data, please ensure that the statement adheres to our policy

RNA-sequencing raw and processed data has been deposited at the University Hamburg Research Data Repository (https://www.fdr.uni-hamburg.de/) with the following identifiers: 10.25592/uhhfdm.8358 and 10.25592/uhhfdm.8372
The mass-spectrometry-based proteomics data were deposited at the ProteomeXchange Consortium (http://proteomecentral.proteomexchange.org) via the PRIDE partner repository with the following dataset identifiers: PXD022789.

Field-specific reporting

Please select the one below that is the best fit for your research. If you are not sure, read the appropriate sections before making your selection.

☒ Life sciences ☐ Behavioural & social sciences ☐ Ecological, evolutionary & environmental sciences

For a reference copy of the document with all sections, see nature.com/documents/nr-reporting-summary-flat.pdf

Life sciences study design

All studies must disclose on these points even when the disclosure is negative.

Sample size	No statistical method were used to determine sample size as they were chosen based on availability (unique autopsy material) and previous experience for omics analyses, enabling us to analyse at least n=5 for each experimental group.
Data exclusions	No data was excluded.
Replication	Experiments were replicated as indicated in each figure legend.
Randomization	Not relevant to this study, as this is an observational and not an interventional study
Blinding	Blinding was used whenever possible (ie. validation of IF/ISH vs RT-qPCR in tissues, pathology examination). However, in most experiments blinding was not possible as analytical groups were defined a priori (ie. omics, where samples were selected into specific categories)

Reporting for specific materials, systems and methods

We require information from authors about some types of materials, experimental systems and methods used in many studies. Here, indicate whether each material, system or method listed is relevant to your study. If you are not sure if a list item applies to your research, read the appropriate section before selecting a response.

Materials & experimental systems

n/a	Involved in the study	
☐	☒	Antibodies
☒	☐	Eukaryotic cell lines
☒	☐	Palaeontology and archaeology
☒	☐	Animals and other organisms
☐	☒	Human research participants
☒	☐	Clinical data
☒	☐	Dual use research of concern

Methods

n/a	Involved in the study	
☒	☐	ChIP-seq
☒	☐	Flow cytometry
☒	☐	MRI-based neuroimaging

Antibodies

Antibodies used	ACE2 (R&D systems, AF933, dilution 1:200)<br>SARS-CoV SΔ10 within S2 domain protein (Genetex, GTX632604, dilution 1:200)<br>SR-B1 (Abcam, ab217318, dilution 1:200)
Validation	ACE2 was validated using human kidney tissues, showing strong expression in the proximal tubuli.<br>SARS-CoV SΔ10 within S2 domain protein (Genetex, GTX632604) was validated in previous experiments (Puelles et al NEJM 2020 and Braun et al Lancet 2020) using infected Vero cells as positive controls and normal human tissue as negative controls.<br>SR-B1 was validated comparing the expression pattern to the report of Wei, C., et al. HDL-scavenger receptor B type 1 facilitates SARS-CoV-2 entry. Nat Metab. 2(12):1391-1400. doi: 10.1038/s42255-020-00324-0 (2020).

Human research participants

Policy information about studies involving human research participants

Population characteristics	De-identified clinical data from two independent clinical cohorts was included - detailed information can be obtained from Fig. 1, Table S1, Table S2, and Data Source File 1.<br>The rest of the study was performed on autopsy tissue samples. Details can be obtained in Fig. 2 and Table S3
Recruitment	For clinical cohorts, patient data was acquired using an institutional board waiver of inform consent, meaning there was no active recruitment, rather clinical data collection of all hospitalized patients due to COVID-19 or diagnosis of COVID-19 during hospitalization. All available data was used, minimizing bias introduction.<br>For autopsy cohorts, we included tissues of patients with confirmed COVID-19 diagnosis.

Ethics oversight	Data collection from clinical patient cohorts were approved by the Institutional Review Board (IRB) of the University of Michigan (HUM00178971) and Hamburg (WF-052/20). The IRB approved a waiver of informed consent for this observational study. No compensation was provided.<br>For the autopsy study, the Ethics Committee of the Hamburg Chamber of Physicians was informed about the study (2020-10353-BO-ff and PV7311). Informed consent was obtained from a next of kin or legal representatives for autopsy and tissue sampling. No compensation was paid.

Note that full information on the approval of the study protocol must also be provided in the manuscript.
