# Supplementary figures and images for: Molecular consequences of SARS-CoV-2 liver tropism
Source: Nat Metab. 2022 Mar 28;4(3):310–9. doi: 10.1038/s42255-022-00552-6 (PMC8964418; doi:10.1038/s42255-022-00552-6)

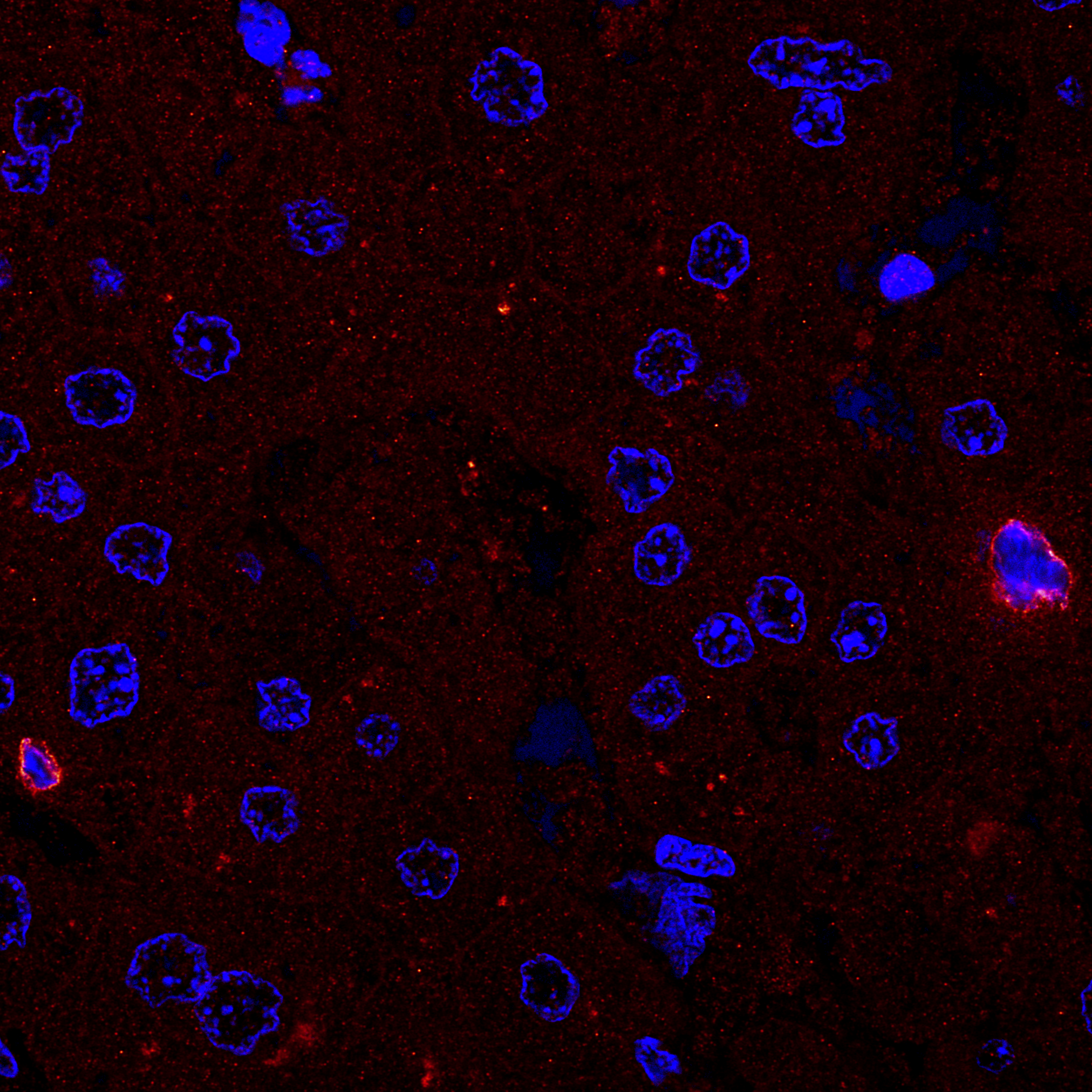

Supplement: Source Data Fig. 2 — ACE2 and SARS-CoV-2 spike microscopy images. [file 42255_2022_552_MOESM5_ESM.zip › F2C left.tif]

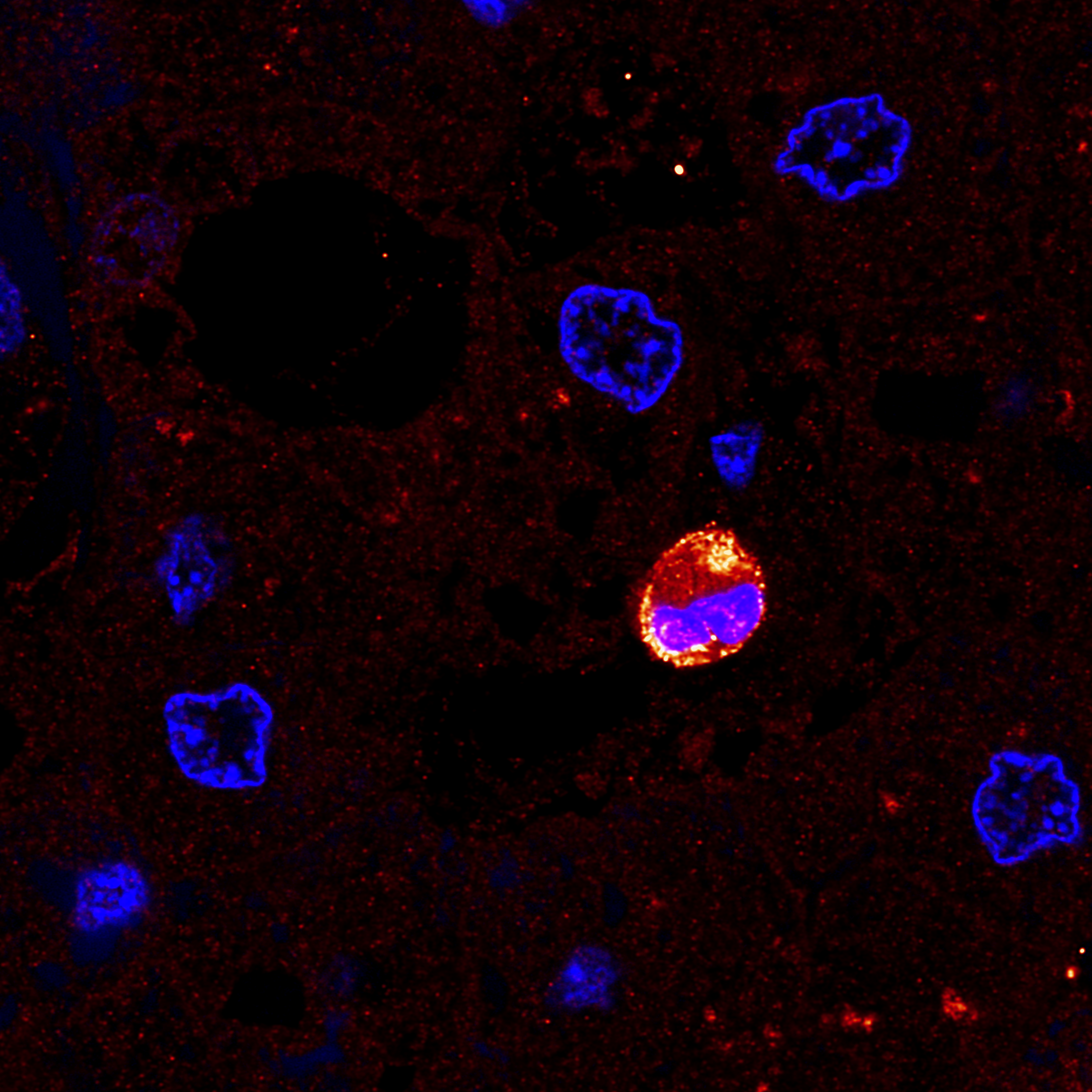

Supplement: Source Data Fig. 2 — ACE2 and SARS-CoV-2 spike microscopy images. [file 42255_2022_552_MOESM5_ESM.zip › F2C right.tif]

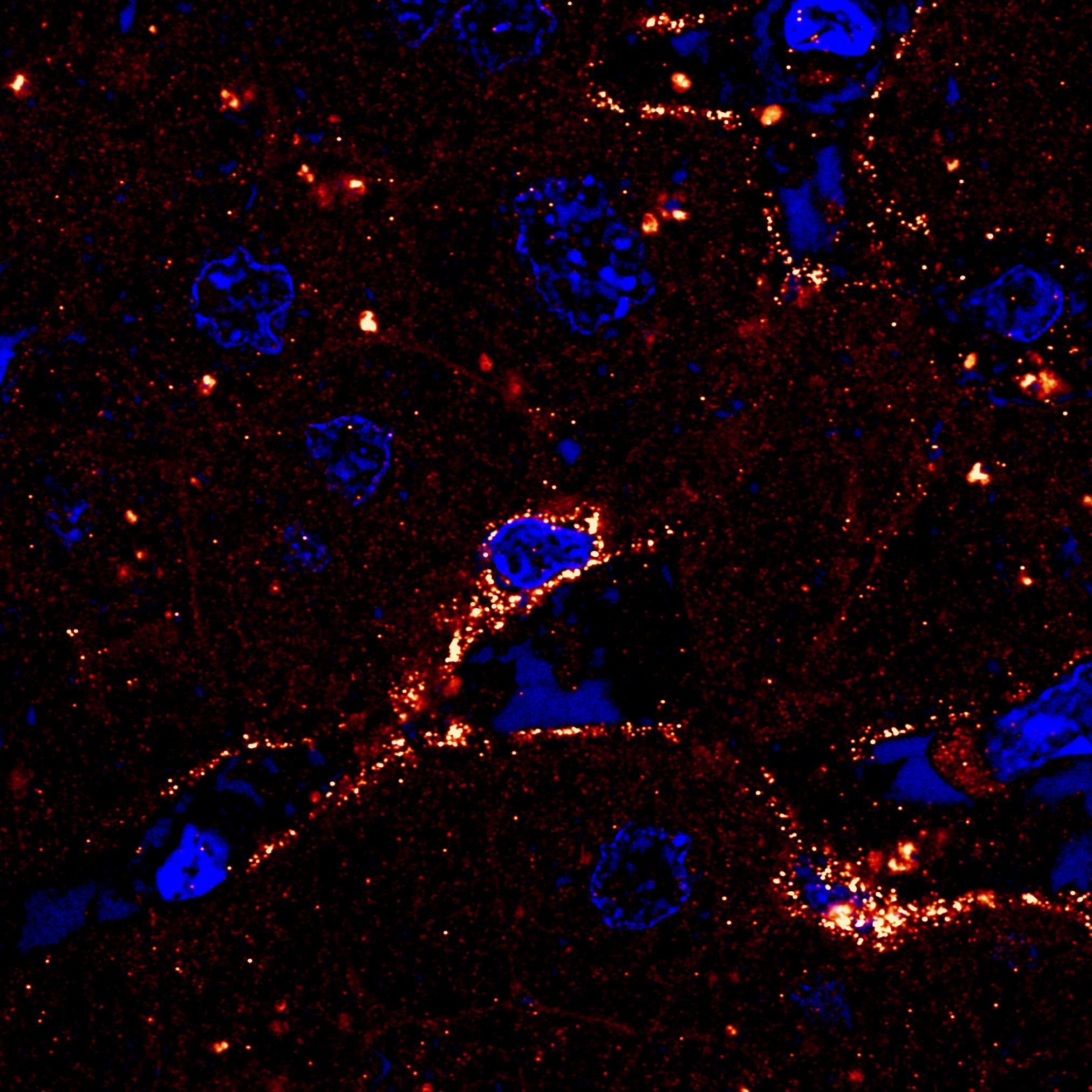

Supplement: Source Data Fig. 2 — ACE2 and SARS-CoV-2 spike microscopy images. [file 42255_2022_552_MOESM5_ESM.zip › Fig2B.tif]

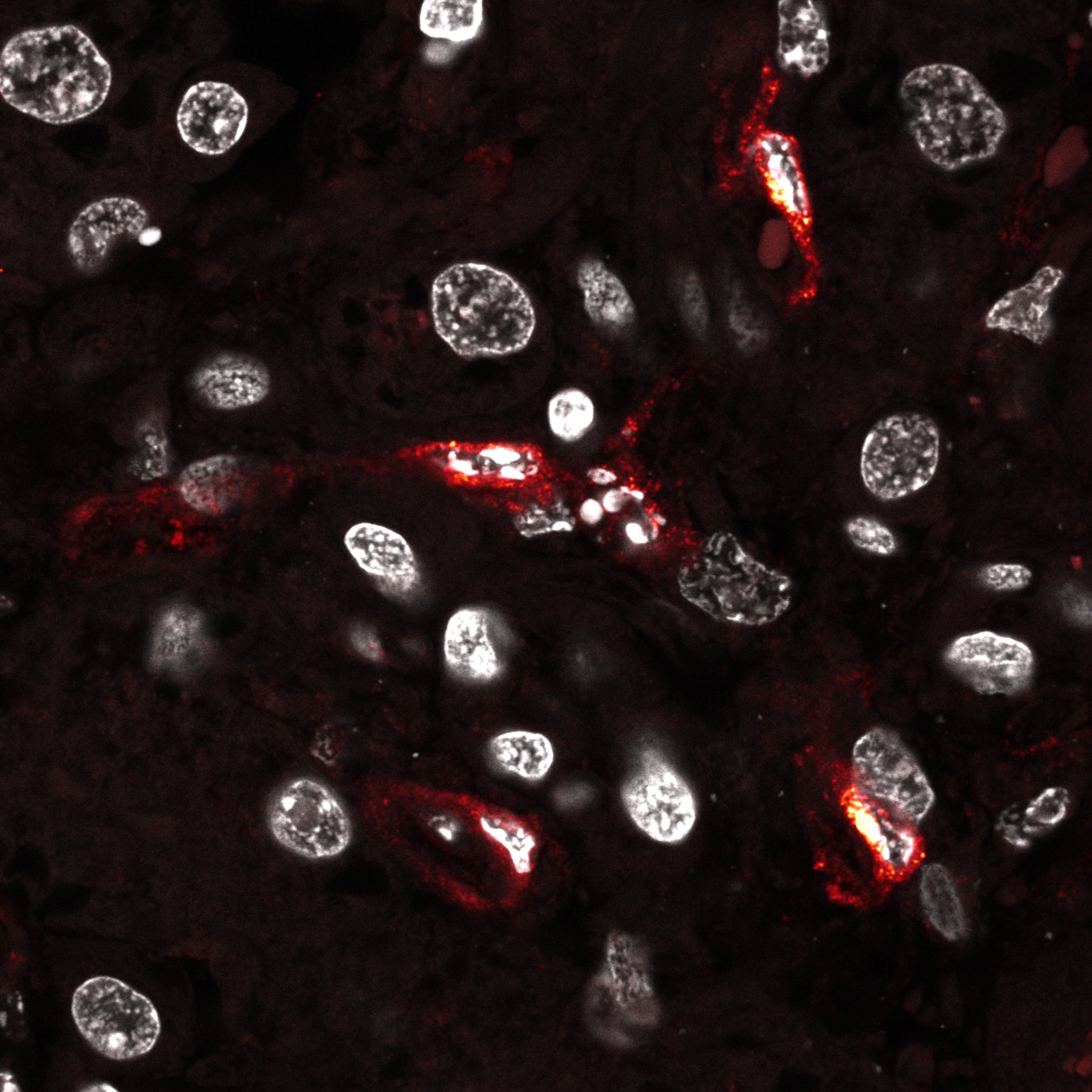

Supplement: Source Data Extended Data Fig. 2 — ACE2 microscopy images. [file 42255_2022_552_MOESM7_ESM.zip › ACE2 Patient 1.jpg]

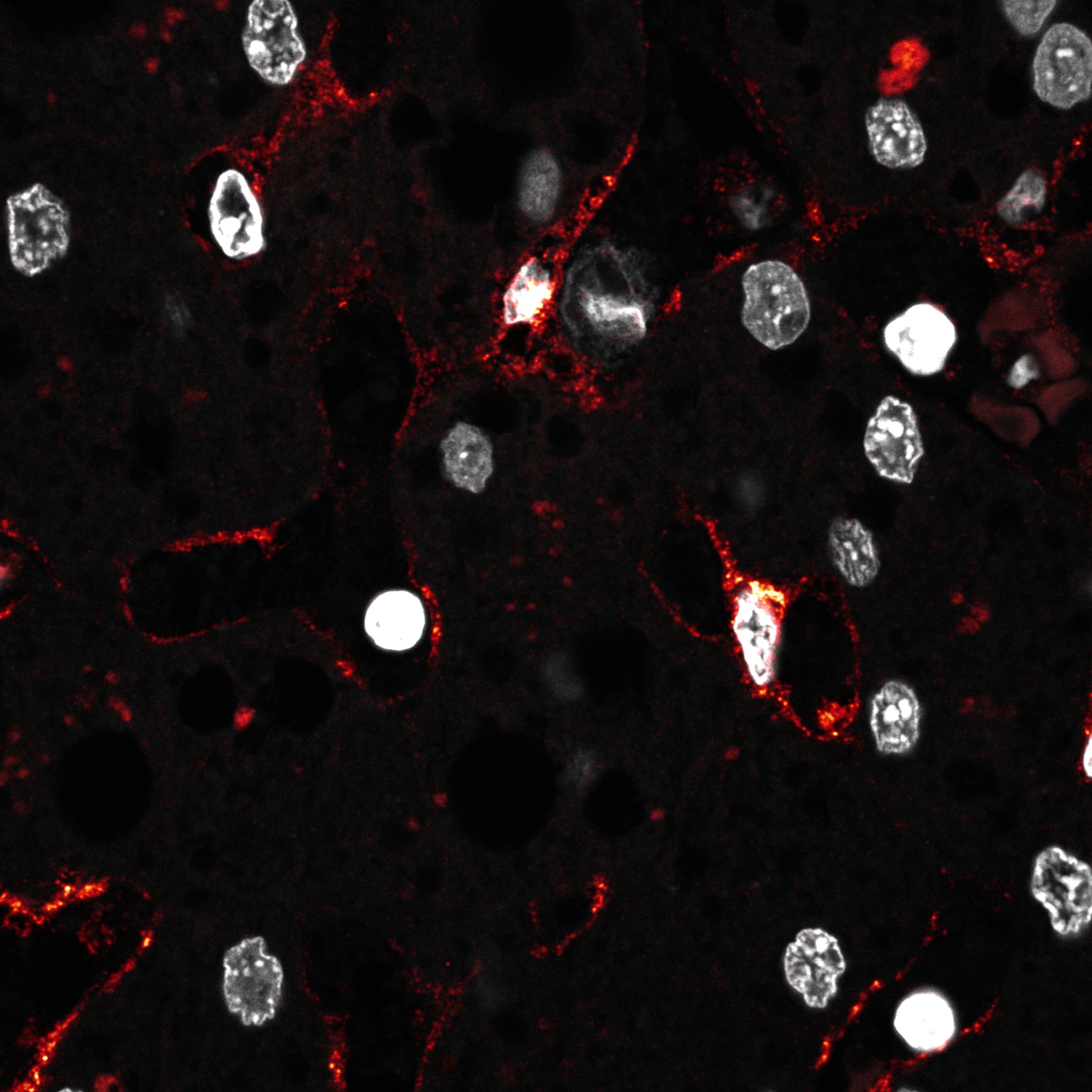

Supplement: Source Data Extended Data Fig. 2 — ACE2 microscopy images. [file 42255_2022_552_MOESM7_ESM.zip › ACE2 Patient 2.jpg]

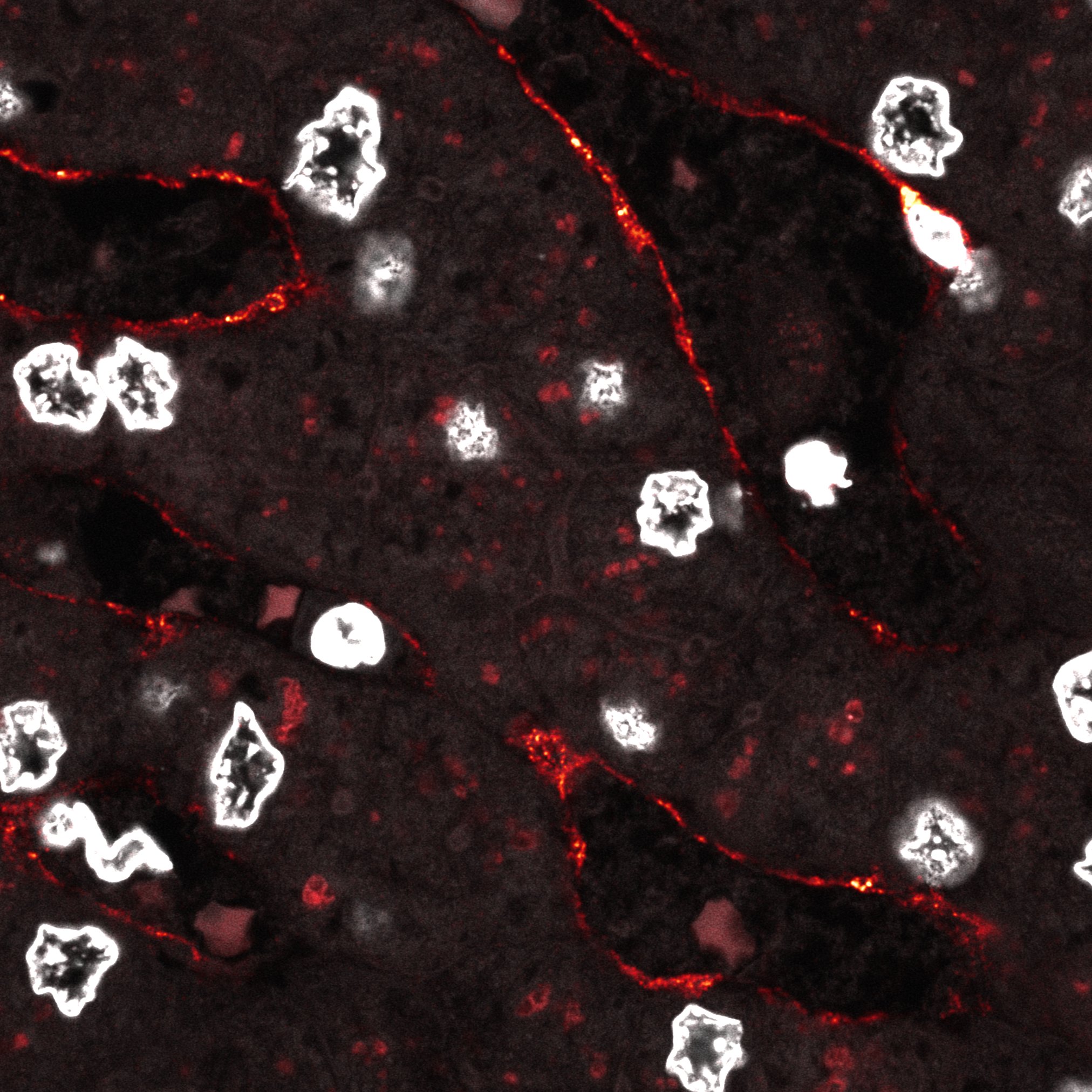

Supplement: Source Data Extended Data Fig. 2 — ACE2 microscopy images. [file 42255_2022_552_MOESM7_ESM.zip › ACE2 Patient 3.jpg]

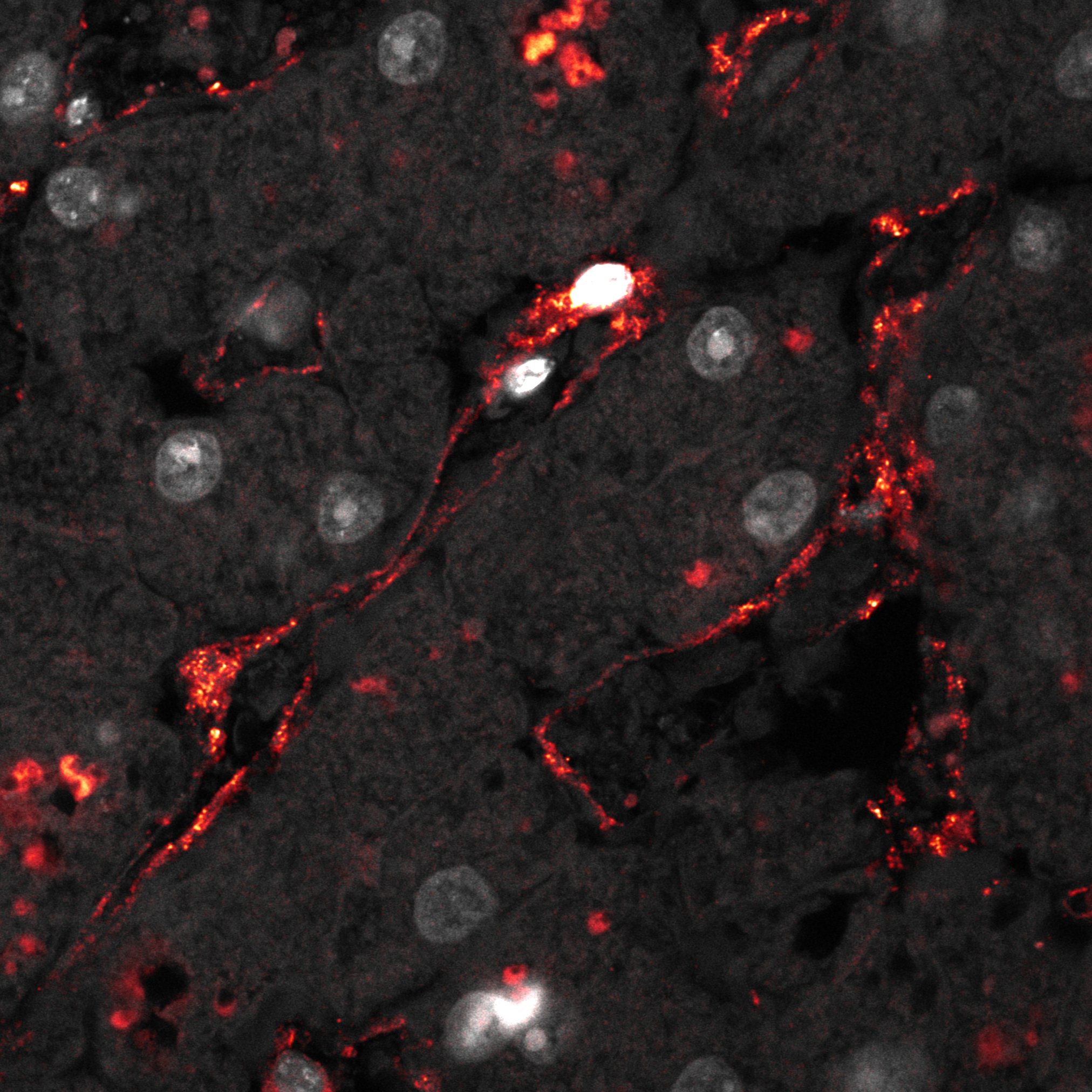

Supplement: Source Data Extended Data Fig. 2 — ACE2 microscopy images. [file 42255_2022_552_MOESM7_ESM.zip › ACE2 Patient 4.jpg]

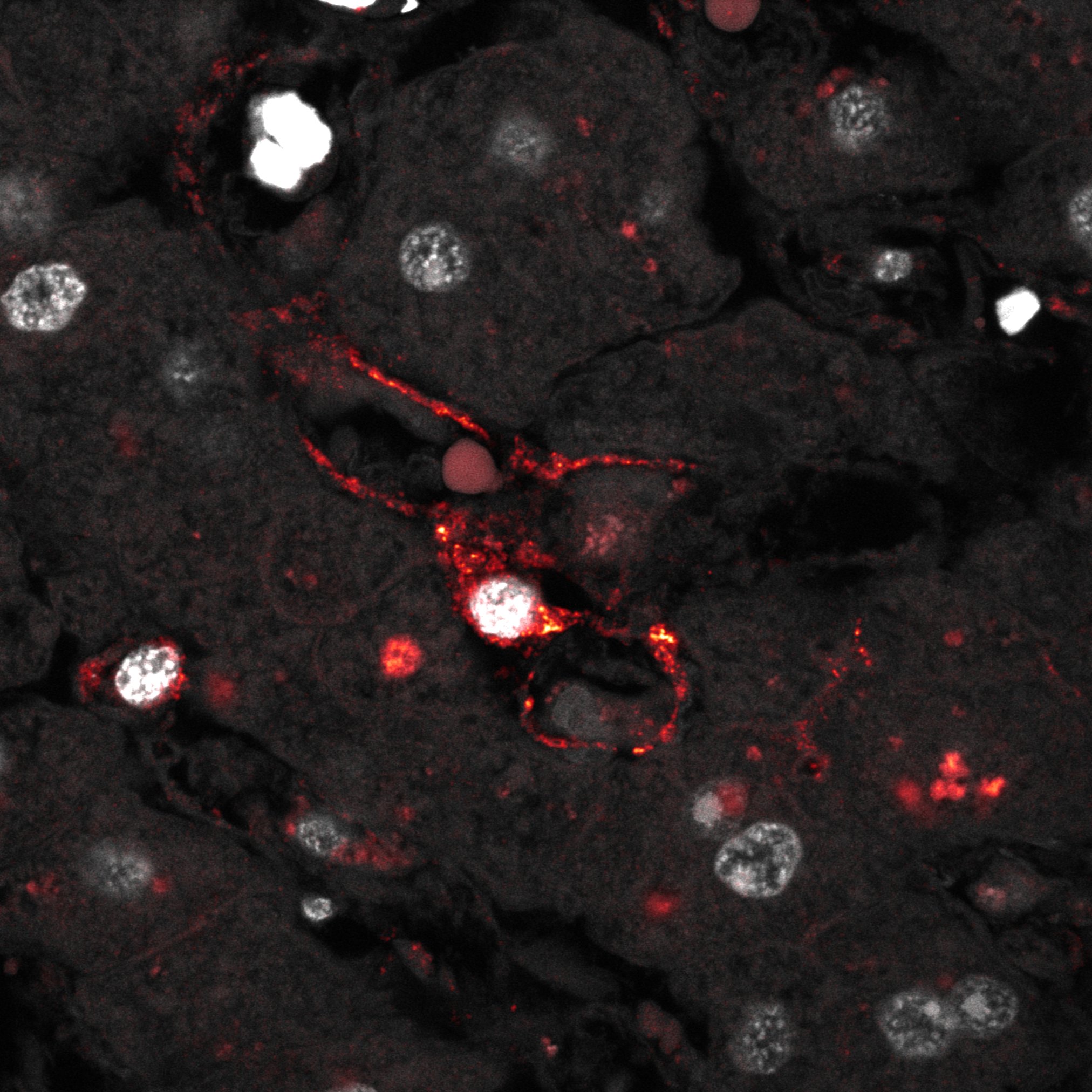

Supplement: Source Data Extended Data Fig. 2 — ACE2 microscopy images. [file 42255_2022_552_MOESM7_ESM.zip › ACE2 Patient 5.jpg]

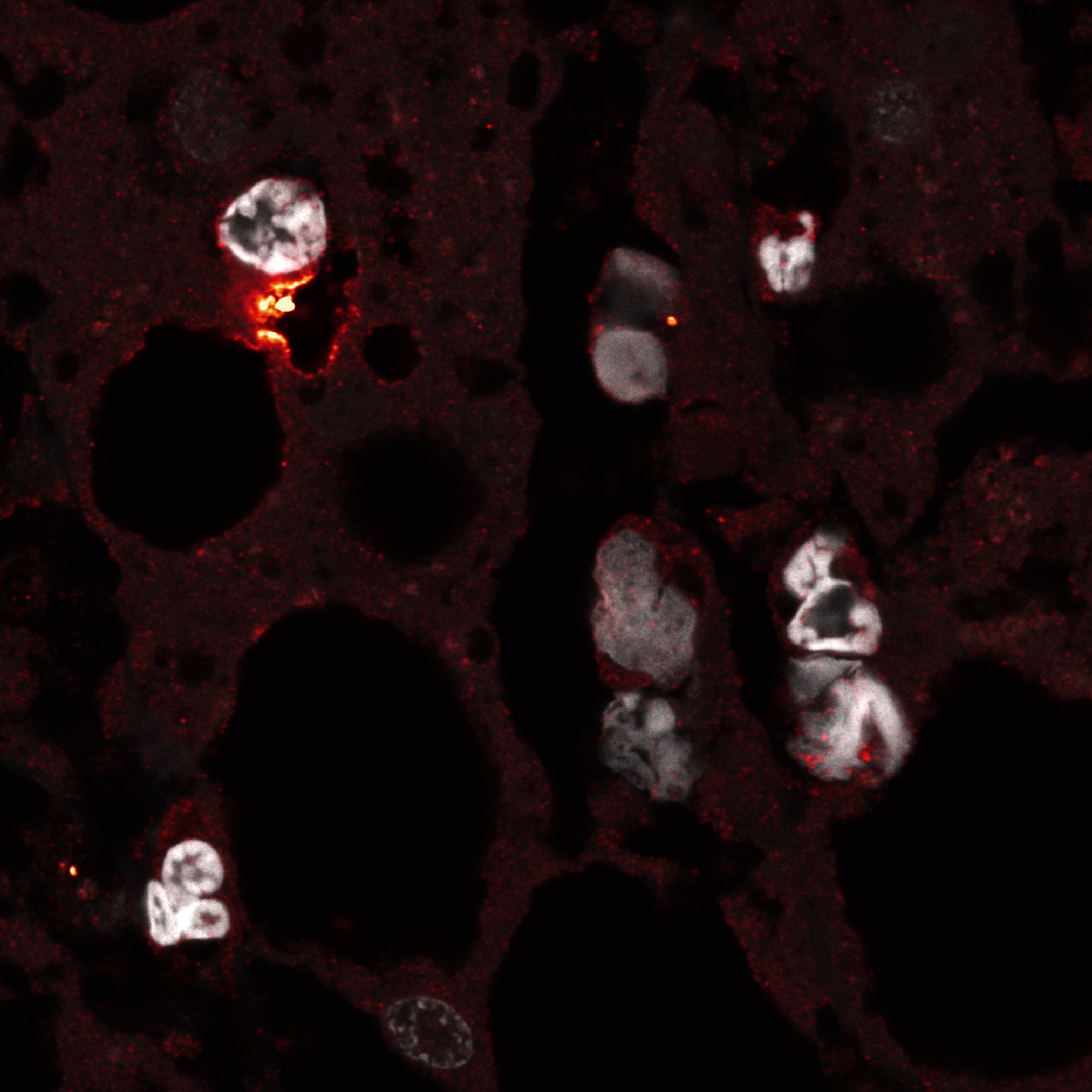

Supplement: Source Data Extended Data Fig. 3 — SARS-CoV-2 spike microscopy images. [file 42255_2022_552_MOESM8_ESM.zip › Spike Patient 1 a1.jpg]

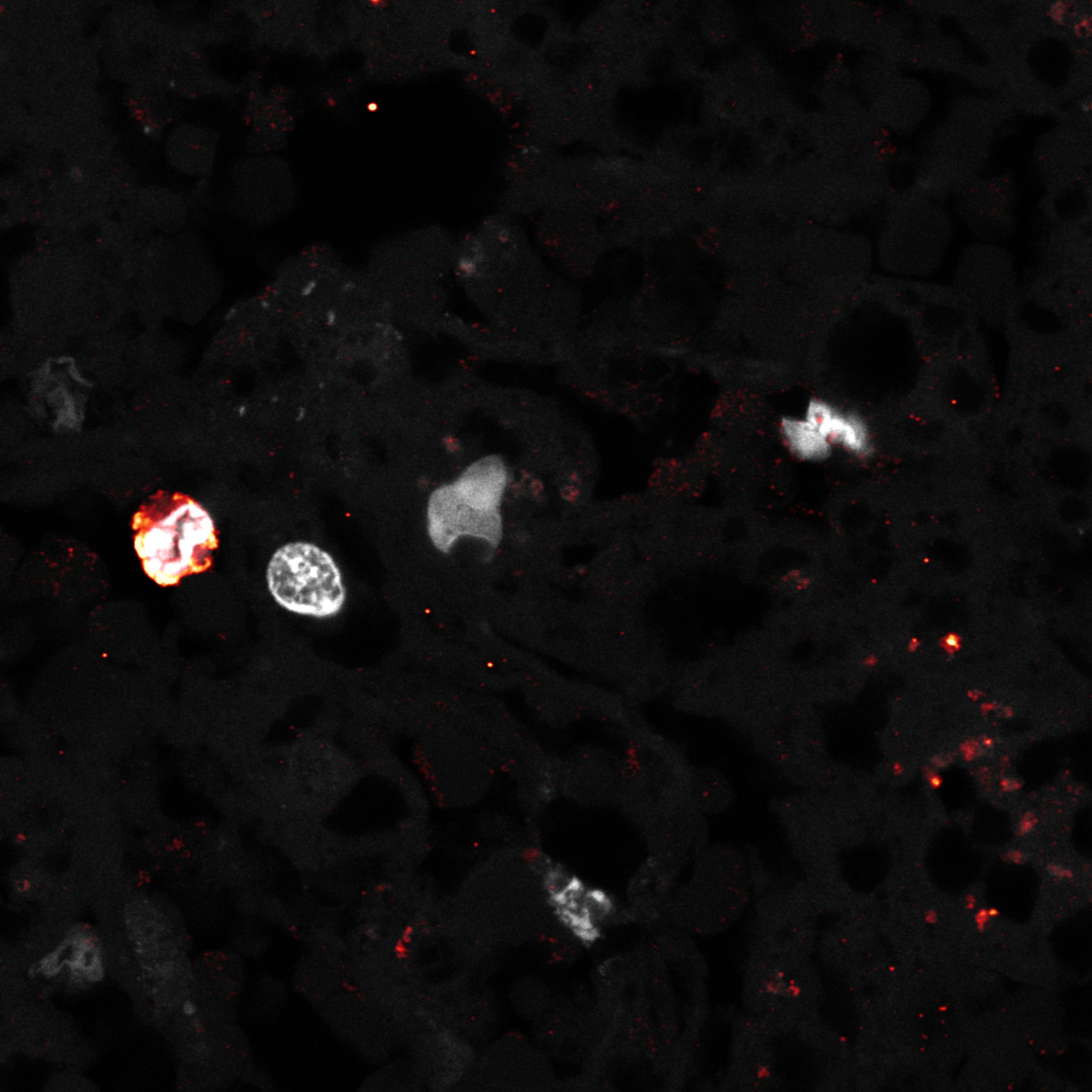

Supplement: Source Data Extended Data Fig. 3 — SARS-CoV-2 spike microscopy images. [file 42255_2022_552_MOESM8_ESM.zip › Spike Patient 1 a2.jpg]

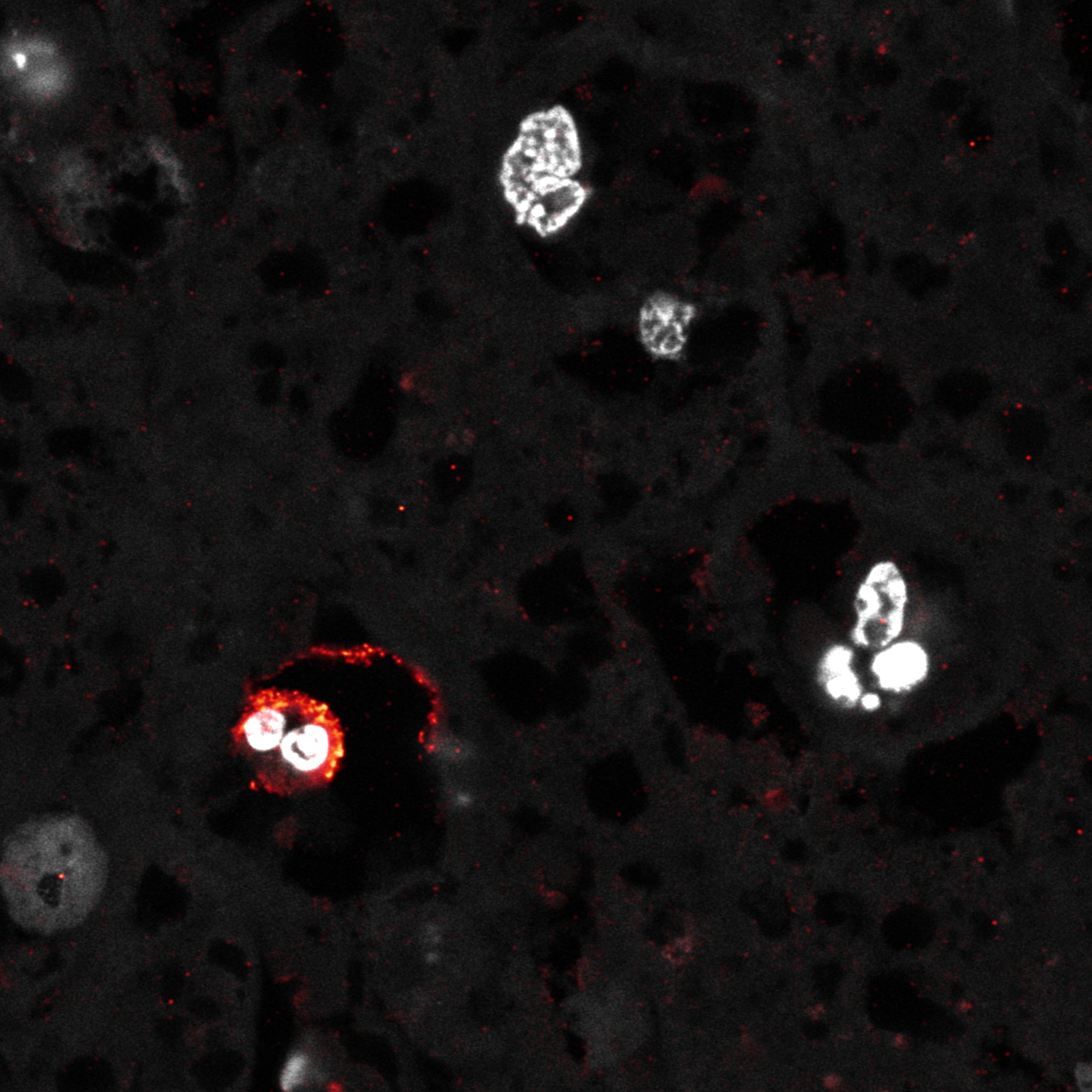

Supplement: Source Data Extended Data Fig. 3 — SARS-CoV-2 spike microscopy images. [file 42255_2022_552_MOESM8_ESM.zip › Spike Patient 1 a3.jpg]

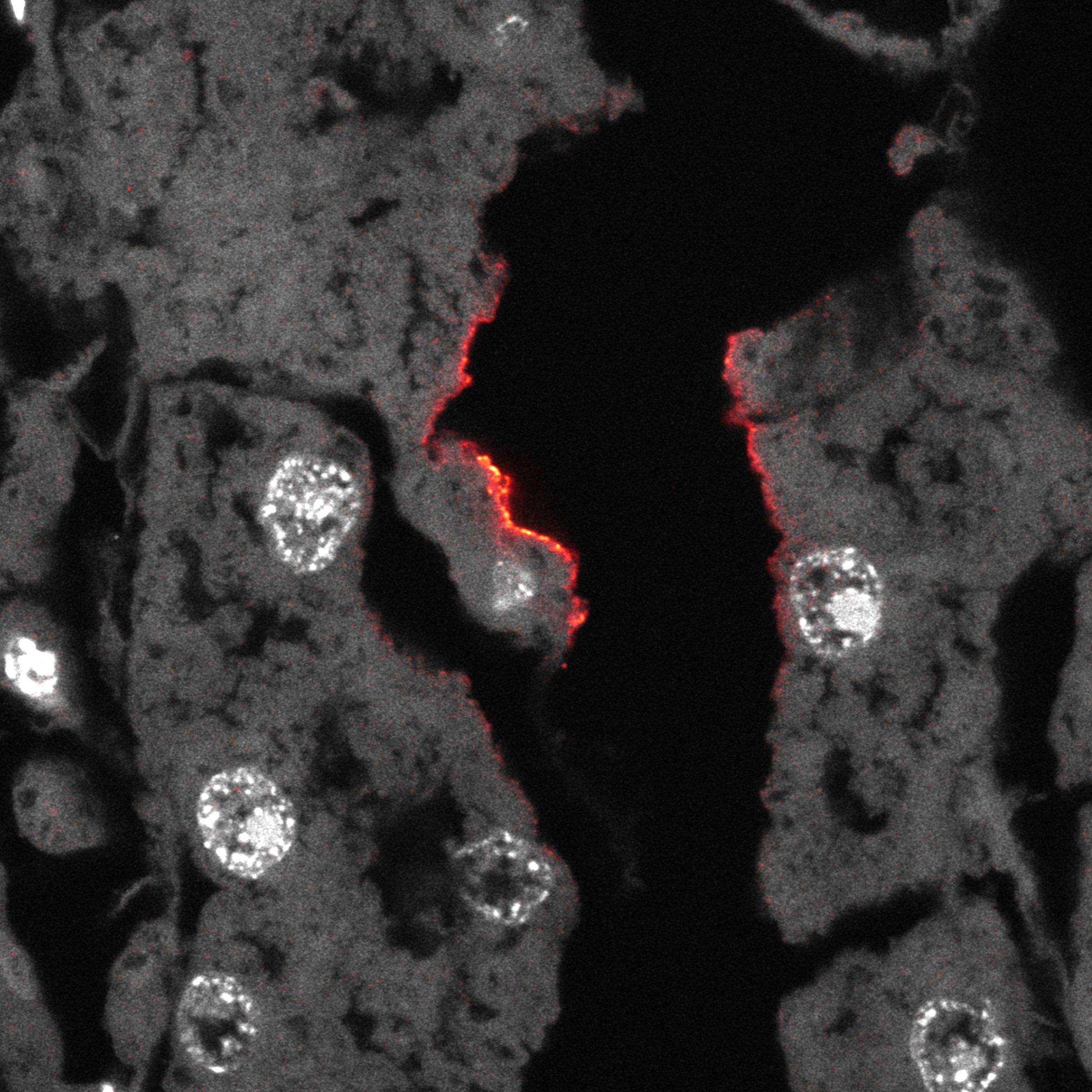

Supplement: Source Data Extended Data Fig. 3 — SARS-CoV-2 spike microscopy images. [file 42255_2022_552_MOESM8_ESM.zip › Spike Patient 2 a1.jpg]

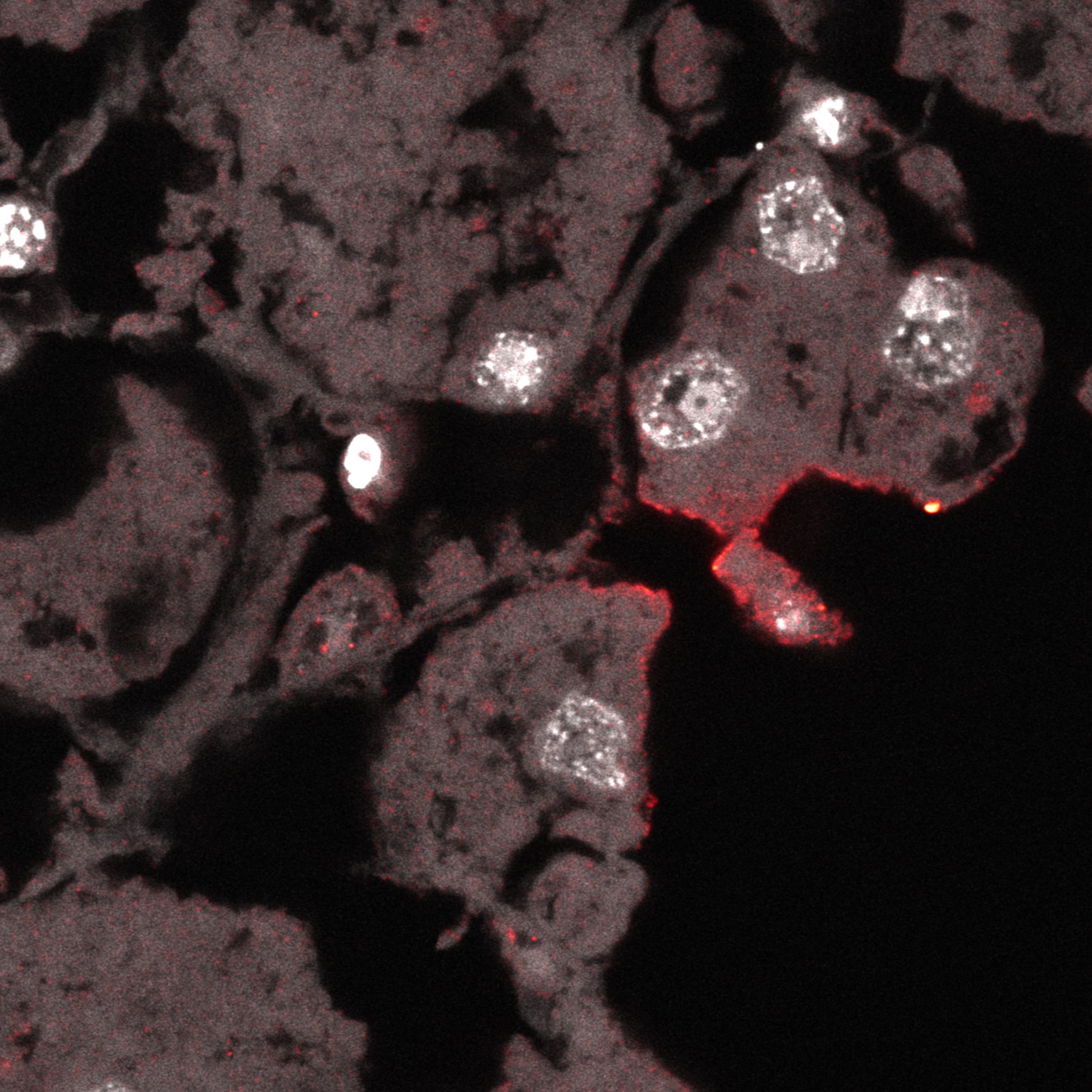

Supplement: Source Data Extended Data Fig. 3 — SARS-CoV-2 spike microscopy images. [file 42255_2022_552_MOESM8_ESM.zip › Spike Patient 2 a2.jpg]

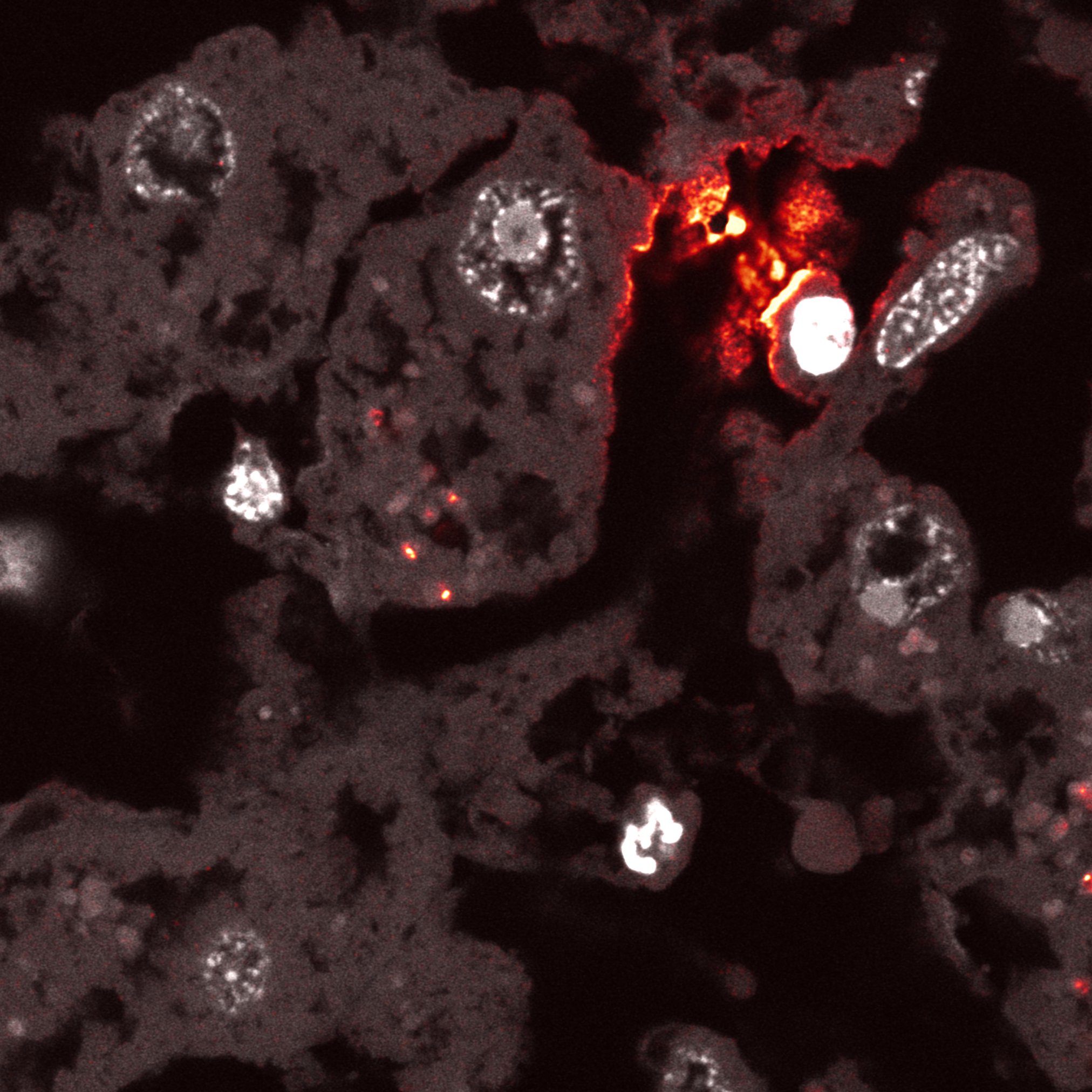

Supplement: Source Data Extended Data Fig. 3 — SARS-CoV-2 spike microscopy images. [file 42255_2022_552_MOESM8_ESM.zip › Spike Patient 2 a3.jpg]

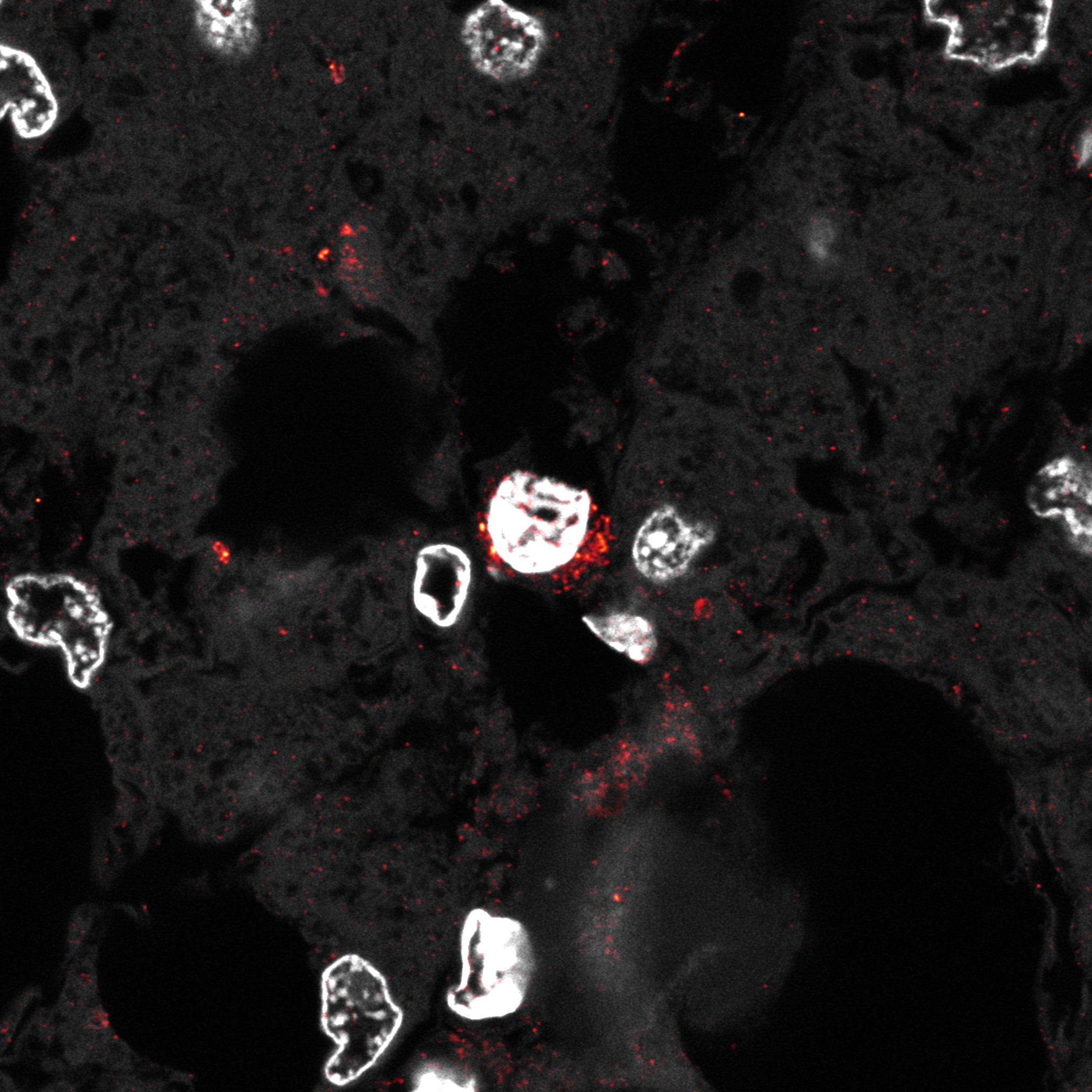

Supplement: Source Data Extended Data Fig. 3 — SARS-CoV-2 spike microscopy images. [file 42255_2022_552_MOESM8_ESM.zip › Spike Patient 3 a1.jpg]

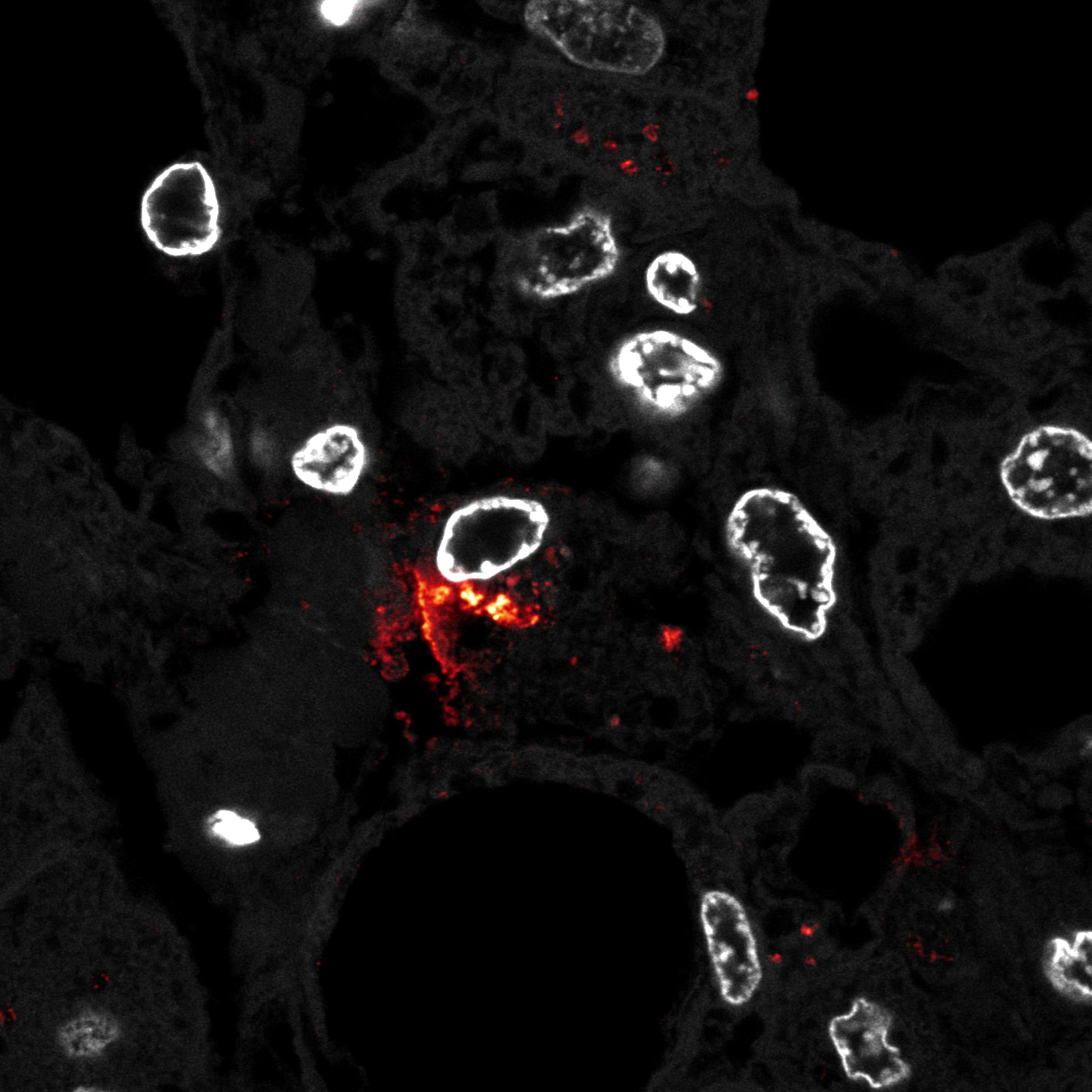

Supplement: Source Data Extended Data Fig. 3 — SARS-CoV-2 spike microscopy images. [file 42255_2022_552_MOESM8_ESM.zip › Spike Patient 3 a2.jpg]

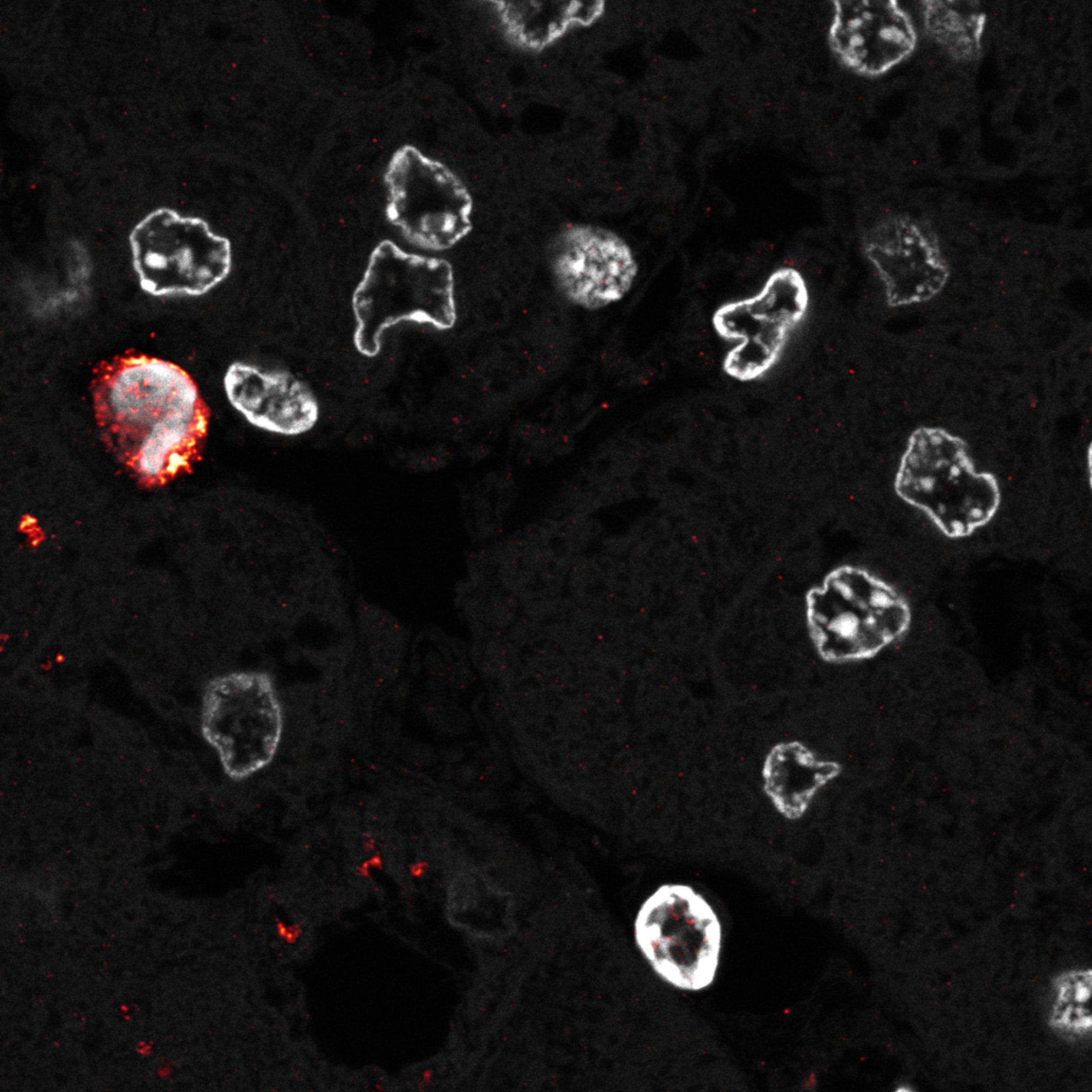

Supplement: Source Data Extended Data Fig. 3 — SARS-CoV-2 spike microscopy images. [file 42255_2022_552_MOESM8_ESM.zip › Spike Patient 3 a3.jpg]

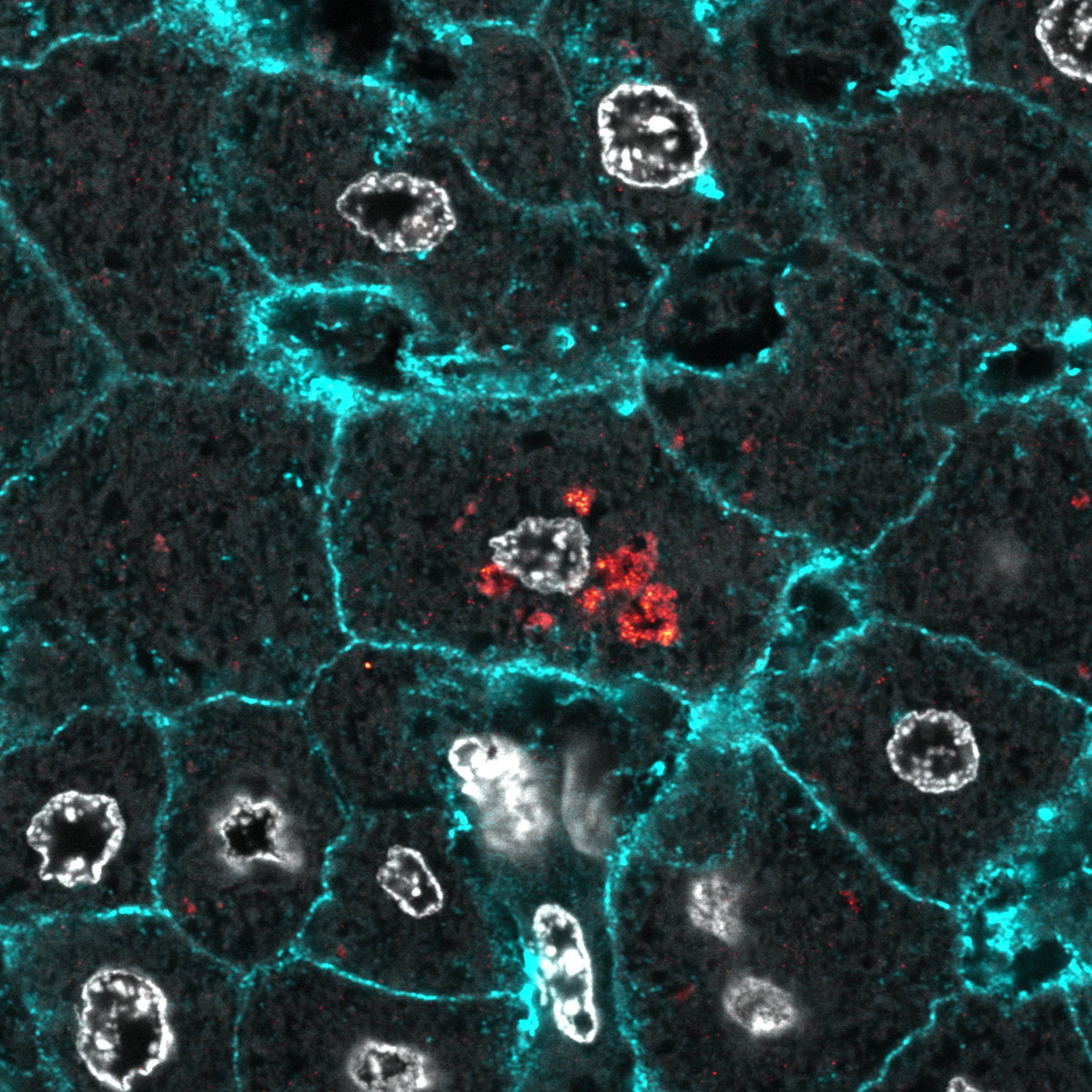

Supplement: Source Data Extended Data Fig. 10 — SRB1 and SARS-CoV-2 spike microscopy images. [file 42255_2022_552_MOESM10_ESM.zip › SR-B1 Patient 1 a1.jpg]

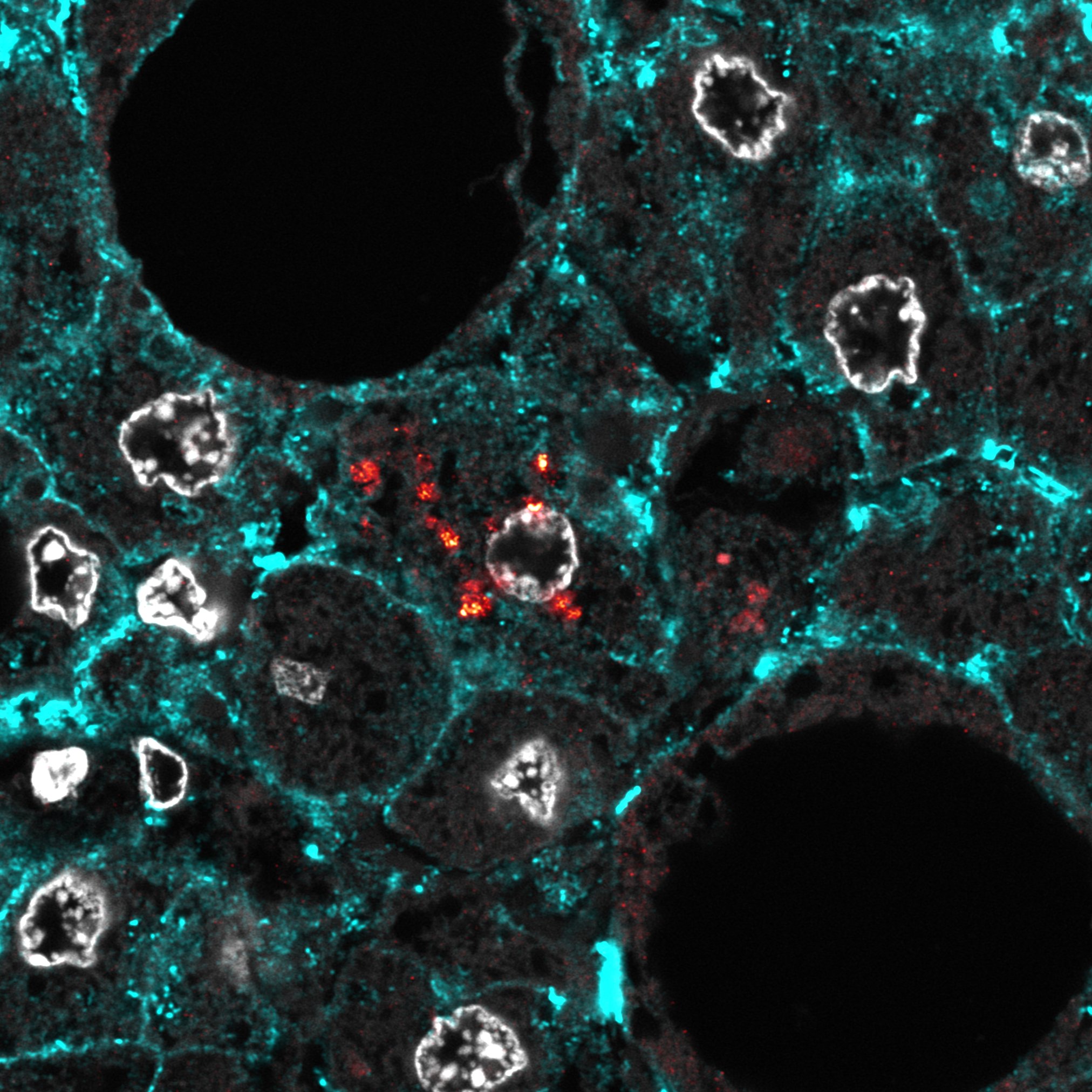

Supplement: Source Data Extended Data Fig. 10 — SRB1 and SARS-CoV-2 spike microscopy images. [file 42255_2022_552_MOESM10_ESM.zip › SR-B1 Patient 1 a2.jpg]

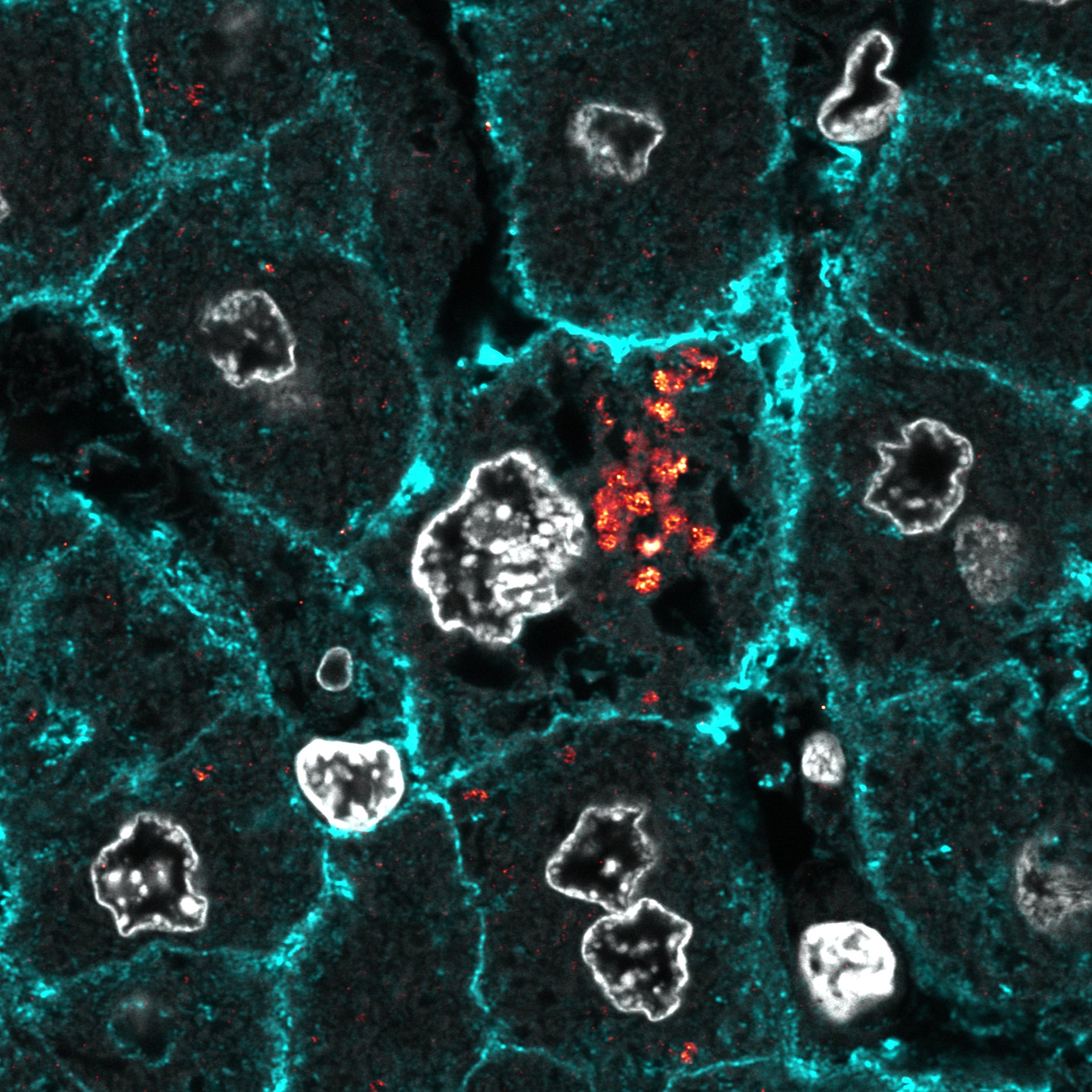

Supplement: Source Data Extended Data Fig. 10 — SRB1 and SARS-CoV-2 spike microscopy images. [file 42255_2022_552_MOESM10_ESM.zip › SR-B1 Patient 1 a3.jpg]

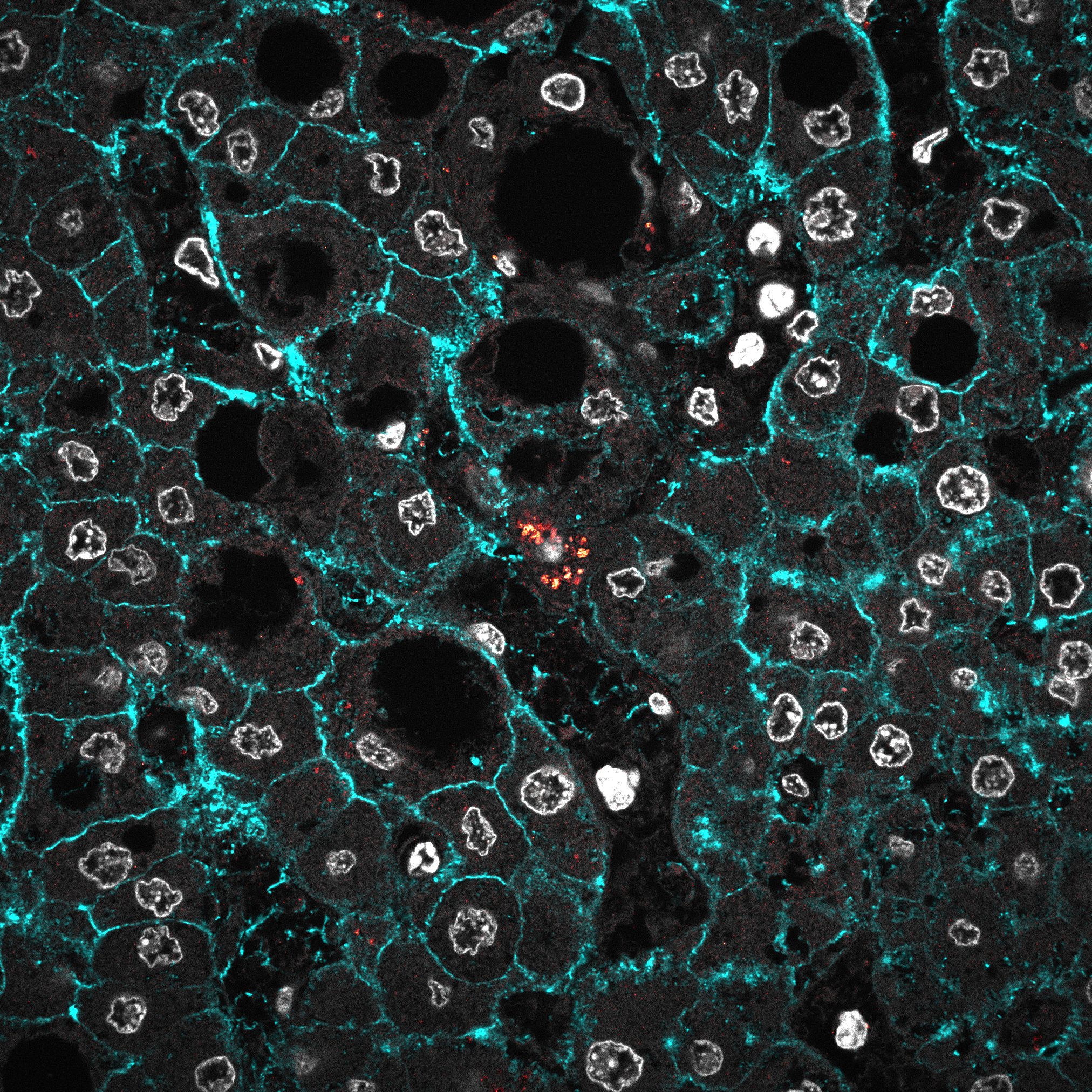

Supplement: Source Data Extended Data Fig. 10 — SRB1 and SARS-CoV-2 spike microscopy images. [file 42255_2022_552_MOESM10_ESM.zip › SR-B1 Patient 1 a4 overview.jpg]

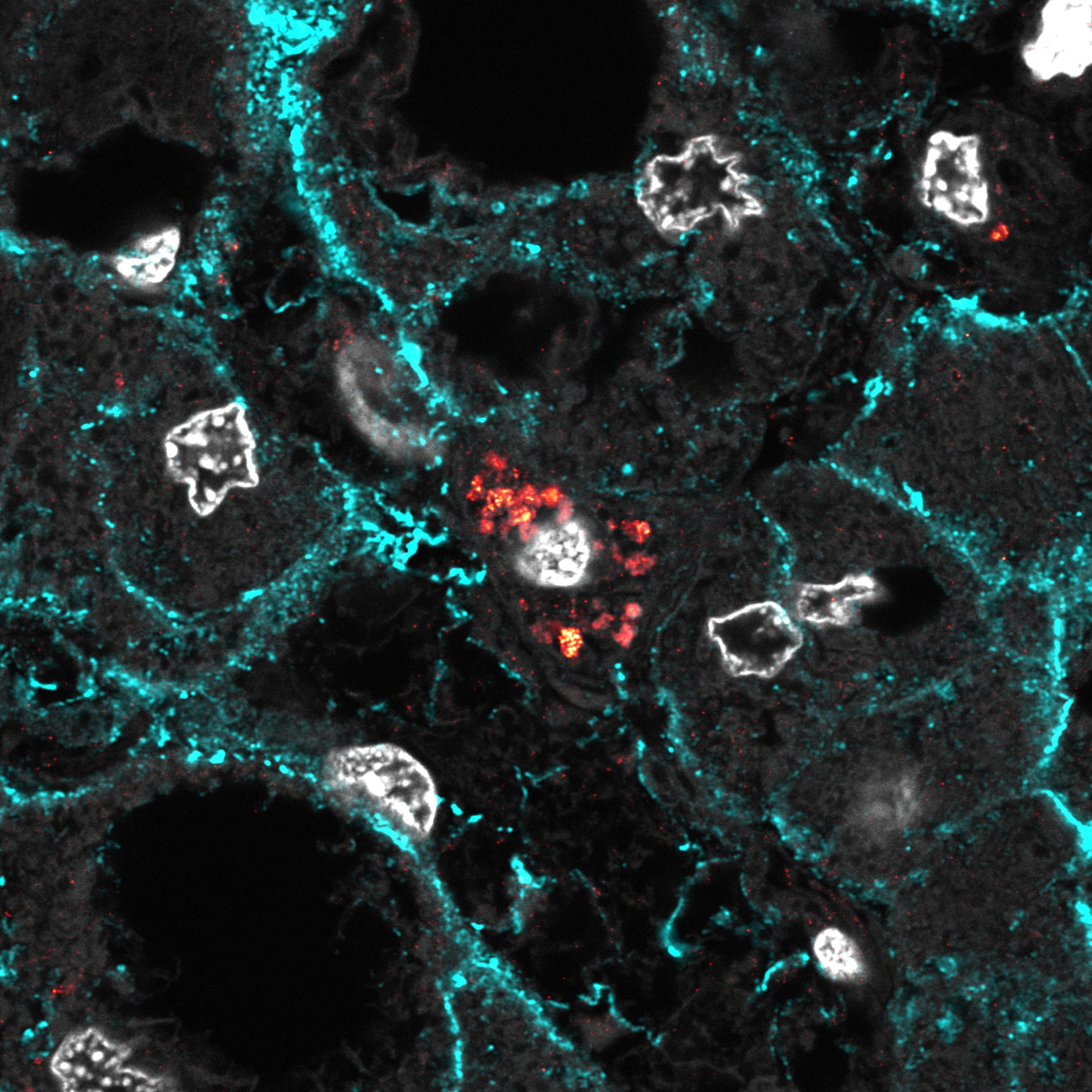

Supplement: Source Data Extended Data Fig. 10 — SRB1 and SARS-CoV-2 spike microscopy images. [file 42255_2022_552_MOESM10_ESM.zip › SR-B1 Patient 1 a4 zoom.jpg]
